# PDIA3 Inhibition Facilitates Sensitivity of IKE‐Induced Ferroptosis via STAT3/LCN2 Axis to Improve Glioblastoma Therapy

**DOI:** 10.1002/advs.202514191

**Published:** 2025-12-14

**Authors:** Jie Zhang, Wei Wang, Xin Liu, Peifen Lu, Xinjing Liu, Qiucheng Nie, Siyuan Xin, Hong Li, Donglin Yu, Xinyue Zhang, Kailong Li, Xiaomin Han, Shuping Zhang, Wei Chong, Lili Sun, Wei Li, Tao Xin, Jin Jiao, Qiang Ma, Yiju Wei

**Affiliations:** ^1^ Biomedical Sciences College & Shandong Medicinal Biotechnology Center Shandong First Medical University & Shandong Academy of Medical Science Jinan Shandong 250117 China; ^2^ Cancer Biology Institute Baotou Medical College Inner Mongolia Autonomous Region Baotou 014040 China; ^3^ Medical Science and Technology Innovation Center Shandong First Medical University & Shandong Academy of Medical Science Jinan Shandong 250117 China; ^4^ School of Life Science Shandong First Medical University & Shandong Academy of Medical Science Tai'an Shandong 271016 China; ^5^ Department of Biochemistry and Biophysics Beijing Key Laboratory of Protein Posttranslational Modifications and Cell Function School of Basic Medical Sciences Peking University Health Science Center Beijing 100191 China; ^6^ Department of Gastrointestinal Surgery Shandong Provincial Hospital Affiliated to Shandong First Medical University Jinan Shandong 250117 China; ^7^ Division of Hematology and Oncology Department of Pediatrics Penn State Cancer Institute Penn State College of Medicine Hershey PA 17033 USA; ^8^ Department of Neurosurgery The First Affiliated Hospital of Shandong First Medical University and Shandong Provincial Qianfoshan Hospital Jinan 250117 China

**Keywords:** ferroptosis, glioblastoma, LCN2, NEDD4L, PDIA3

## Abstract

Ferroptosis, an iron‐dependent form of programmed cell death, has emerged as a promising therapeutic approach in glioblastoma (GBM). Nonetheless, the role and mechanism governing vulnerability to ferroptosis in GBM have remained unknown. In this study, we identify protein disulfide isomerase A3 (PDIA3) as a crucial factor mediating the vulnerability of glioma cells to ferroptosis and demonstrate that inhibition or depletion of PDIA3 enhances IKE‐induced ferroptosis in GBM cells. Mechanistically, NEDD4L functions as an E3 ubiquitin ligase to promote ferroptosis by facilitating K29‐linked ubiquitination of PDIA3 via its C‐terminal HECT domain. Furthermore, NEDD4L‐mediated ubiquitination of PDIA3 enhances ferroptosis by downregulating the expression of LCN2 through its interaction with STAT3 independently of ATF4. Here, a drug delivery system is presented using a tetrahedral DNA nanostructure (TDN) encapsulating IKE (TDN‐IKE) to penetrate the blood–brain barrier. The combined use of TDN‐IKE and PDIA3 inhibitors exhibits a synergistic antitumor effect against GBM therapy in vivo, providing a potential therapeutic approach for ferroptosis‐based therapy in GBM. Overall, these findings demonstrate a novel mechanism by which PDIA3 regulates ferroptosis, indicating that a promising therapeutic strategy for GBM is through inhibiting of SLC7A11 and PDIA3.

## Graphical abstract

In this manuscript, protein disulfide isomerase A3 (PDIA3) is identified as a key factor mediating the susceptibility of ferroptosis in GBM. Inhibition of PDIA3 enhances IKE or cystine starvation‐induced ferroptosis in GBM cells by resulting in the accumulation of lipid peroxidation and a reduction in GSH level. Mechanistically, NEDD4L functions as an E3 ubiquitin ligase to promote ferroptosis by facilitating K29‐linked ubiquitination of PDIA3 via its C‐terminal HECT domain. PDIA3 suppresses ferroptosis by regulating the expression of LCN2 through its interaction with STAT3. Consequently, A drug delivery system is established by using a tetrahedral DNA nanostructure (TDN) encapsulating IKE (TDN‐IKE) for penetrating the blood–brain barrier (BBB). The combination of TDN‐IKE and PDIA3 inhibition exhibits a synergistic antitumor effect for GBM therapy in vivo, providing a potential therapeutic approach for ferroptosis‐based therapy in GBM.

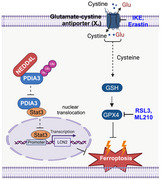

## 1 Introduction

Glioblastoma (GBM), a form of grade IV glioma, is the most lethal and most common primary brain cancer in adults. It is highly prone to recurrence and significantly affects the survival and quality of life of patients, with a 5‐year survival rate of less than 5%.^[^ ^1^ ^]^ In recent decades, the primary treatment mode for patients with GBM has involved a combination of surgery and chemoradiotherapy; however, these approaches have shown limited efficacy, resulting in an urgent need for new treatment modalities.^[^ ^2^ ^]^ Emerging evidence suggests that GBM cells exhibit dysregulated iron metabolism during tumor growth and invasion, which renders them more susceptible to ferroptosis than other tumor cells.

Ferroptosis is a novel form of cell death that differs from apoptosis and other regulated cell death types in that it involves the accumulation of oxidized phospholipids resulting from oxidative damage caused by iron ions, which in turn results in cell membrane rupture and cell death.^[^ ^3^ ^]^ Multiple pathways regulating ferroptosis have been reported, including mechanisms involving the SLC7A11/GPX4 (glutathione peroxidase 4) axis, FSP1‐CoQ10, the GCH1/BH4‐phospholipid axis, and MBOAT1/2 and monounsaturated fatty acids (MUFAs).^[^ ^4^, ^5^, ^6^, ^7^, ^8^, ^9^ ^]^ Thus, targeting ferroptosis has emerged as an effective anti‐tumor approach that could be used to induce cancer cell death via inhibition of specific target molecules, such as SLC7A11, GPX4, and FSP1, with potential applications in the treatment of patients with GBM.^[^ ^10^, ^11^, ^12^, ^13^, ^14^, ^15^, ^16^ ^]^ For instance, a recent study demonstrated that a combination of temozolomide, a drug used to treat GBM, with erastin, an inducer of ferroptosis, could enhance therapeutic efficacy against glioma cells.^[^ ^17^ ^]^ However, little is known about the molecular pathways that underpin this synergistic effect.^[^ ^18^ ^]^ The role of ferroptosis in the initiation and progression of glioma and the underlying mechanisms and key molecular processes involved have remained largely unexplored. Therefore, more research is necessary to determine precisely how target molecules regulate ferroptosis vulnerability.

The protein disulfide isomerase (PDI) family contains a series of endoplasmic reticulum (ER)‐resident thioredoxin proteases and chaperones that play important roles in maintaining cellular homeostasis by mediating oxidative protein folding.^[^ ^19^ ^]^ This family consists of 21 members, primarily located in the ER, with some also present in other cellular compartments, including the cell surface, cytosol, mitochondria, and nucleus. Levels of PDIs are elevated in various cancers and have been implicated in particular in proliferation, invasion, and metastasis of cancers including glioma,^[^ ^20^, ^21^ ^]^ ovarian cancer,^[^ ^22^ ^]^ prostate cancer,^[^ ^23^ ^]^ and lung cancer.^[^ ^24^ ^]^ Therefore, PDIs are regarded as novel targets in cancer therapy. A robust relationship exists between PDI expression and clinical outcomes in glioma, with P4HB (PDIA1), PDIA4, and PDIA5 in particular showing promise for prediction of patient survival and tumor progression.^[^ ^25^ ^]^ Notably, PDIA3 (protein disulfide isomerase A3) exhibits redox regulation and isomerase activity in the ER.^[^ ^26^ ^]^ It is considered a potential pharmacological target in GBM, where it is highly expressed and has diverse functions in cancer progression and immune‐related cancer treatment.^[^ ^25^, ^27^ ^]^ SUMOylation, a post‐translational modification involving the small ubiquitin‐like modifier (SUMO), of PDIA3 modulates its enzymatic activity, protein‐binding ability, and ER localization, thereby mediating ER stress and STAT3 (signal transducer and activator of transcription 3) activation in pancreatic beta cells.^[^ ^28^ ^]^ PDIA3 further interacts with NF‐ĸB and STAT3 in the nuclear or mammalian target of rapamycin in the cytoplasm. Clinical studies have also shown that targeting PDIA3 to suppress its expression or activity shows promise as a therapy against cancer,^[^ ^29^ ^]^ autoimmune diseases,^[^ ^30^ ^]^ obesity^[^ ^31^ ^]^ and metabolic disorders.^[^ ^32^, ^33^ ^]^ Targeting PDIA3 is thus a potential means of overcoming resistance to ferroptosis in GBM; however, its molecular mechanism remains to be explored.

Herein, we used a bioinformatic and small interfering RNA (siRNA) screening approach to identify PDIA3 as a crucial PDI family member mediating the resistance of glioma cells to ferroptosis. We found that PDIA3 stability is regulated by E3 ubiquitin ligase NEDD4L and is essential to IKE‐induced ferroptosis. PDIA3 mediates ferroptosis resistance by regulating the STAT3–LCN2 axis. In addition, we developed a novel drug delivery material, tetrahedral DNA nanostructure (TDN), for blood–brain barrier (BBB) permeability and showed that inhibition of PDIA3 combined with IKE treatment could enhance GBM therapy by targeting ferroptosis.

## 2 Results

### 2.1 PDIA3 is a Key PDI Family Gene that Regulates IKE‐Induced Ferroptosis Resistance

**Figure 1:**
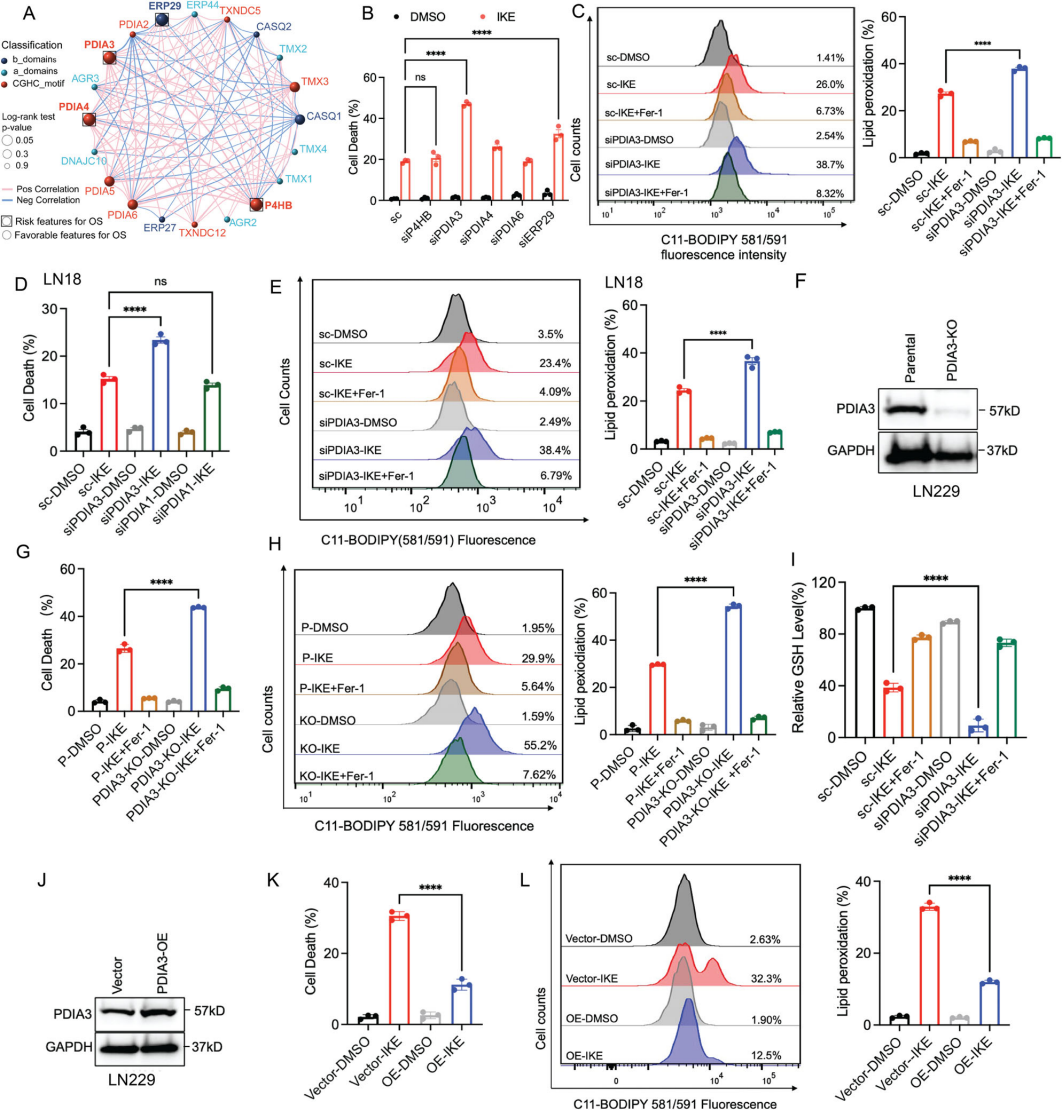
PDIA3 is a crucial PDI family gene that mediates IKE‐ and cystine starvation‐induced ferroptosis. A) TCGA database analysis of associations between PDI family genes. B) Detection of cell death using propidium iodide (PI) via flow cytometry in LN229 cells transfected with the indicated siRNAs and scrambled control (sc) under various treatments for 24 h. IKE at 25 µm, Fer‐1 at 1 µm. C) Detection of lipid peroxidation using C11‐BODIPY (581/591) through a flow cytometer in LN229 cells transfected with the indicated siRNAs and sc under treatments for 16 h. IKE at 25 µm, Fer‐1 at 1 µm. D) Detection of cell death among LN18 cells, transfected with the indicated siRNAs under treatments for 24 h (IKE at 25 µm, Fer‐1 at 1 µm). E) Detection of lipid peroxidation using C11‐BODIPY (581/591) via flow cytometry in LN18 cells transfected with indicated siRNAs and sc under treatments for 16 h (IKE at 25 µm, Fer‐1 at 1 µm). F) Detection of cell death using propidium iodide (PI) via flow cytometry among LN229 parental and PDIA3 knock out (KO) cells under IKE treatment with or without Fer‐1 treatments for 24 h. G) Detection of lipid peroxidation using C11‐BODIPY (581/591) in LN229 parenta(P) and PDIA3‐KO cells under IKE treatment with or without Fer‐1 treatments for 16 h. H) Immunoblot analysis of whole cell lysates (WCL) obtained from PDIA3‐KO and LN229 parental cells. I) Detection of glutathione (GSH) levels in LN229 cells transfected with the indicated siRNAs and sc under IKE treatment with or without Fer‐1 treatments for 24 h. J) Immunoblot analysis of whole‐cell lysates (WCL) derived from LN229 cells with overexpression (OE) of PDIA3 and control cells transfected with an empty vector. K) Detection of cell death using PI through flow cytometry in LN229 control and PDIA3‐OE cells under IKE treatment with or without Fer‐1 treatments for 16 h. L) Detection of lipid peroxidation using C11‐BODIPY (581/591) in LN229 control and PDIA3‐OE cells under IKE with or without Fer‐1 treatment for 16 h. The results are expressed as the mean ± standard deviation. *p < 0.05, **p < 0.01, ***p < 0.001, ****p < 0.0001. *n *= 3 biological repeats; two‐way analysis of variance. Fer‐1, ferrostatin‐1.

Emerging evidence indicates that PDIs are promising targets for treating GBM progression through regulation of ferroptosis.^[^ ^27^ ^]^ To validate whether there is a functional association between PDIs and GBM progression in glioma patients. we analyzed the GBM dataset from The Cancer Genome Atlas (TCGA) and found positive correlations of expression levels of P4HB, PDIA3, PDIA4, and ERP29 with GBM progression, and close associations of high expression of these genes with poor patient survival (**Figure** **1**A: Figure S1A, Supporting Information). Accumulating studies have demonstrated that PDI family genes are strongly involved in the mediation of chemically induced ferroptosis.^[^ ^34^, ^35^, ^36^, ^37^, ^38^ ^]^ We also found that expression of the aforementioned four genes was positively associated with that of ferroptosis target genes SLC7A11 and GPX4 in GBM patients (Figure S1B,C, Supporting Information). To determine which PDI genes were specific regulators of ferroptosis in GBM, we performed an siRNA screen using a PDI siRNA library targeting PDIA5 and PDIA6, as well as P4HB, PDIA3, PDIA4, and ERP29. The siRNAs were transfected into LN229 cells cultured in a six‐well plate, which were subsequently treated with IKE or cystine starvation. We identified PDIA3 was a prominent key gene for IKE and cystine starvation‐induced cell death in LN229 cell line (Figure 1B; Figure S1D,E, Supporting Information), although ERP29 also showed a notable effect. Then, we used the lipid peroxidation‐sensitive dye C11‐BODIPY 581/591 to estimate IKE‐induced lipid peroxidation in LN229 cells transfected with PDIA3 siRNA. We found that the loss of PDIA3 sensitized IKE‐induced lipid peroxidation, compared to the scramble‐siRNA group (Figure 1C). To validate our screening strategy, we showed that depletion of PDIA3 also sensitized LN18 cells transfected with PDIA3 siRNA to IKE‐induced cell death and lipid peroxidation (Figure 1D,E). Moreover, PDIA3 was essential for IKE‐induced ferroptosis in LN18 cells, but PDIA1 was not (Figure 1D, Supporting Information). Notably, depletion of ERP29 contributed to ferroptosis sensitivity of GBM cells but did not enhance the ferroptosis sensitivity mediated by PDIA3/PDI inhibitor LOC14 (Figure S1F,G, Supporting Information). To further examine whether depletion of PDIA3 contributed to ferroptosis sensitivity of GBM cells, we constructed LN229 cells with knockout (KO) of PDIA3 (Figure 1F) and LN18 cells with knockdown (KD) of PDIA3 (Figure S1,H, Supporting Information), and found that KO or KD of PDIA3 also promoted IKE‐induced ferroptosis in GBM (Figure 1G,H; Figure S1I,J, Supporting Information). To confirm whether PDIA3 affects IKE intracellular glutathione (GSH) level, we observed that depletion of PDIA3 did not affect intracellular GSH but dramatically diminished IKE‐induced GSH level by using siRNAs (Figure 1I). In addition, we constructed LN229 cells with overexpression of PDIA3 and found that PDIA3 overexpression prevented IKE‐induced cell death and lipid peroxidation in GBM (Figure 1J–L). Taken together, these results indicated that PDIA3 was a potentially essential factor in the regulation of IKE‐induced ferroptosis in GBM; however, further work was needed to clarify its regulatory mechanism.

### 2.2 Inhibition of PDIA3 Promotes SLC7A11‐Dependent Ferroptosis in GBM Cells

**Figure 2:**
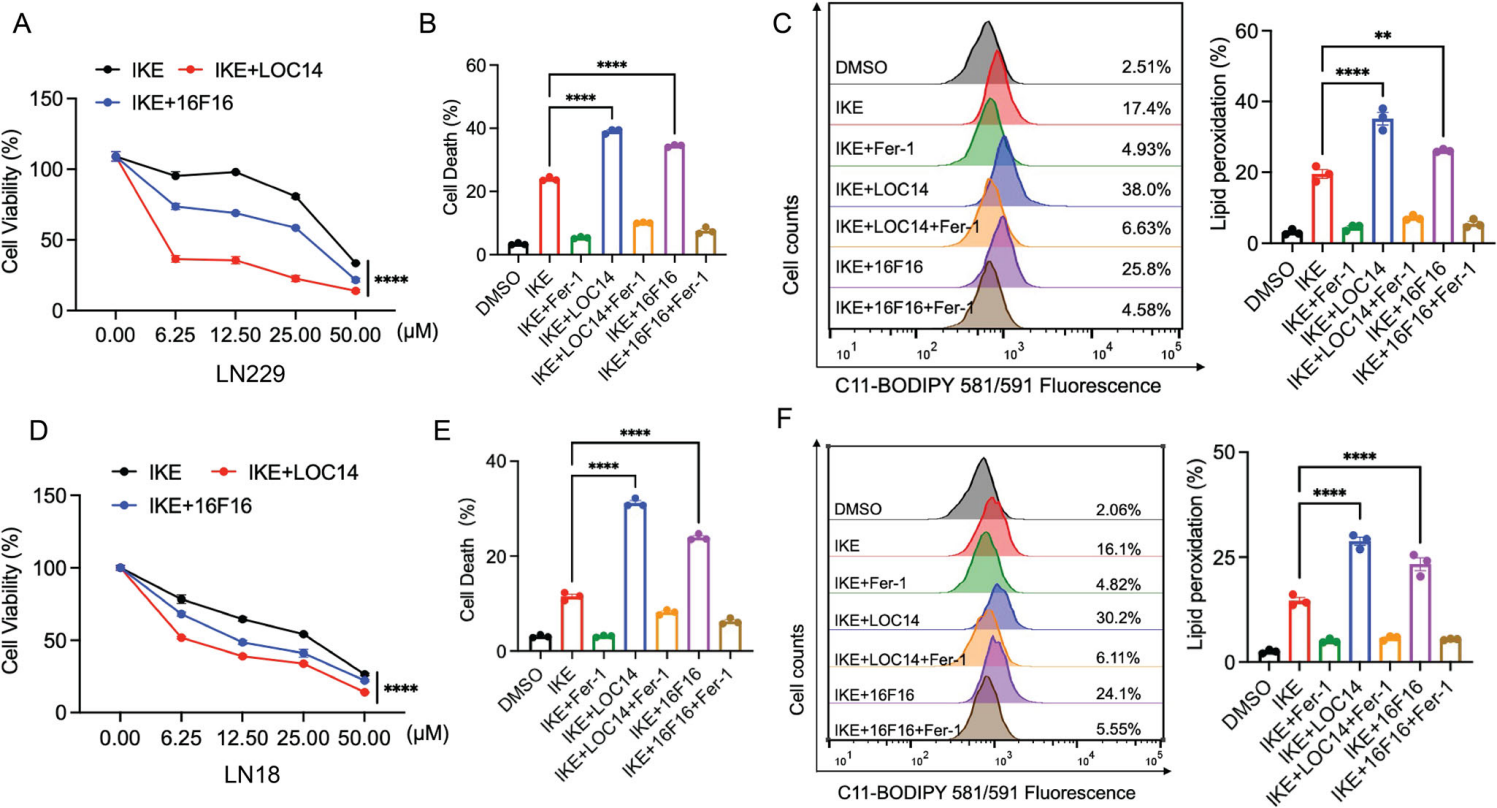
Inhibition of PDIA3 enhances IKE‐induced ferroptosis in GBM. A) Measurement of cell viability of LN229 cells subjected to the indicated treatments for 24 h: IKE (25 µm) and Ferrostatin‐1 (Fer‐1, 1 µm). *n* = 3 for A and D; treatment with PDIA3 inhibitors 16F16 and LOC14 in combination with different concentrations of IKE in LN229 cells. B) Quantitative analysis of cell death by flow cytometry after treatment of LN229 cells with 16F16 and LOC14 in combination with IKE. C) Representative flow cytometry results and quantification of lipid peroxidation levels by flow cytometry after treatment of LN229 cells with 16F16 and LOC14 combined with IKE. D) Cell viability was detected by CCK‐8 after treatment with PDIA3 inhibitors 16F16 and LOC14 in combination with different concentrations of IKE in LN18 cells. E) Quantification of cell death by flow cytometry after treatment of LN18 cells with 16F16 and LOC14 in combination with IKE. F) Representative flow cytometry and quantification of lipid peroxidation levels by flow cytometry after treatment of LN18 cells with 16F16 and LOC14 combined with IKE. The results are expressed as the mean ± standard deviation. **p < 0.01, ****p < 0.0001.

Previous investigations have shown that combination therapies involving PDI inhibitors and anti‐cancer drugs can overcome resistance to chemotherapy via a possible synergistic effect.^[^ ^39^, ^40^, ^41^ ^]^ Nonetheless, it remains unknown whether PDIA3 participates in chemotherapy resistance of GBM by regulating vulnerability to ferroptosis. To investigate the involvement of PDIA3 in ferroptosis and the potential synergetic effects of PDI inhibitors combined with ferroptosis inducers, we assessed cell viability, cell death, and lipid reactive oxygen species (ROS) levels under the combined use of a PDIA3 inhibitor and ferroptosis induction or cystine starvation treatment in GBM cell lines. We found that IKE combined with PDIA3 inhibitor LOC14 or 16F16 both decreased cell viability, with concentration gradient effects compared to IKE treatment alone, and there were obvious synergistic cytotoxic effects in both LN229 and LN18 cells (**Figure** **2**A,D). We next considered whether PDIA3 influenced the induction of ferroptosis by the class II ferroptosis inducer RSL3 through GPX4 inhibition. We found that inhibition of PDIA3 did not affect RSL3‐induced ferroptosis in GBM cell lines treated with LOC14 (Figure S1K,L, Supporting Information). Furthermore, inhibition of PDIA3 not only promotes IKE and cystine starvation‐induced cell death (Figure 2B; Figure S2A, Supporting Information), but also enhances the accumulation of lipid peroxidation (Figure 2C; Figure S2B, Supporting Information) in LN229 cells. Likewise, inhibition of PDIA3 facilitated IKE–induced and cystine starvation–induced cell death (Figure 2E; Figure S2D, Supporting Information), and lipid ROS accumulation (Figure 2F; Figure S2E, Supporting Information) in LN18 cells. However, protein disulfide isomerase PDI (or P4HB) mediates chemically induced GSH depletion‐associated ferroptotic cell death through NOS activation (dimerization) and NO accumulation in hepatocyte cells.^[^ ^38^ ^]^ Notably, we found that inhibition of PDIA3 markedly decreased IKE‐induced intracellular GSH levels, but did not affect intracellular GSH levels after treatment with LOC14 (Figure S2C,F, Supporting Information). These results indicate that PDIA3 inhibition could exert a synergistic effect with SLC7A11 inhibitors in GBM therapy, offering a potential therapeutic approach for ferroptosis‐based therapy in patients with brain tumors.

### 2.3 PDIA3 Stability is Regulated by E3 Ubiquitin Ligase NEDD4L and Necessary for IKE‐Induced Ferroptosis

**Figure 3:**
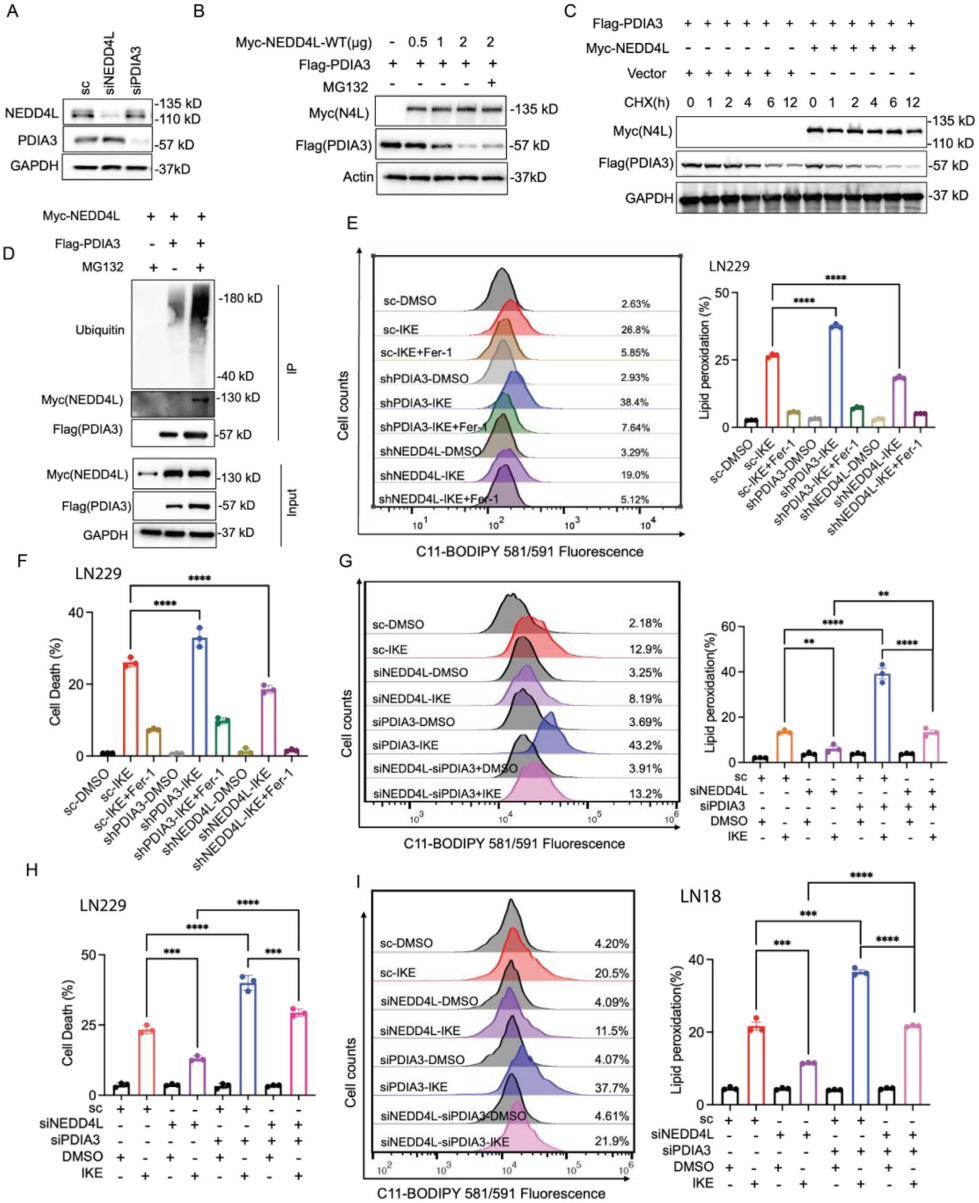
PDIA3 stability is regulated by E3 ubiquitin ligase NEDD4L and is necessary for IKE‐induced ferroptosis. A) Protein level of PDIA3 and NEDD4L in LN229 cells transfected with siRNA, analyzed using western blotting. B) After transfection of different plasmids with 293T, MG132 was used 6 h before sample collection, and the protein expression of Myc and Flag was detected by western blot. C) After transfection of 293T into different plasmids, the protein expression of Myc and Flag was detected by CHX assay according to the time gradient, using western blot analysis. D) Western blot detection of the indicated proteins after transfection of the indicated plasmids in 293T cells. E) Representative flow cytometry results and quantitative analysis of lipid peroxidation levels after IKE treatment in LN229 shPDIA3 and shNEDD4L cells. F) Quantitative analysis of cell death via flow cytometry after IKE treatment in LN229 shPDIA3 and shNEDD4L cells. G) Representative flow cytometry results and quantitative analysis of lipid peroxidation levels after IKE treatment in LN229 (G) and LN18 I) transfected with the indicated siRNAs. H) Quantitative analysis of cell death via flow cytometry after IKE treatment in LN229 cells transfected with the indicated siRNAs. The results are expressed as the mean ± standard deviation. ***p < 0.001, ****p < 0.0001.

We next explored how PDIA3 affects IKE‐induced ferroptosis vulnerability in GBM. First, we speculated that the protein stability of PDIA3 could be related to ferroptosis under IKE treatment. To test this hypothesis, we detected protein levels of PDIA3 when LN229 cells were treated with IKE for different times and observed gradual decreases in PDIA3 protein levels with increasing IKE treatment time (Figure S3A, Supporting Information), while ubiquitination of PDIA3 is dramatically enhanced (Figure S3B, Supporting Information), indicating that PDIA3 proteins were unstable under IKE treatment. We then hypothesized that it could be an E3 ubiquitination ligase of PDIA3, as indicated by mass spectrometry data in a previous study,^[^ ^42^ ^]^ and aimed to verify whether the protein level of PDIA3 required for IKE‐induced ferroptosis was regulated by NEDD4L. We first observed that protein but not mRNA levels of *PDIA3* were increased in LN229 cells treated with NEDD4L siRNA compared to controls (**Figure** **3**A; Figure S3C, Supporting Information). We then considered whether the ubiquitination of PDIA3 relied on the expression of NEDD4L and found that an increase in NEDD4L protein levels led to a reduction in PDIA3 protein levels (Figure 3B). Moreover, the ubiquitination of PDIA3 mediated by NEDD4L was effectively reversed by MG132 (Figure 3B), and expression of ectopic NEDD4L inhibited the quantity of PDIA3 in a dose‐dependent manner. Importantly, the half‐life of PDIA3 protein was markedly shortened following NEDD4L overexpression, and this was accompanied by an increase in PDIA3 ubiquitination (Figure 3C,D). To further investigate whether NEDD4L mediates the proteasomal or autophagic‐lysosomal degradation of PDIA3, we conducted cycloheximide (CHX) assays with MG132 and Bafilomycin A1 (BafA1) treatments. Our observations revealed that BafA1 treatment did not extend the half‐life of PDIA3 protein following MG132 treatment, suggesting that NEDD4L‐mediated degradation of PDIA3 is predominantly reliant on the proteasomal pathway rather than the autophagic‐lysosomal pathway (Figure S3D, Supporting Information). Additionally, we found that KD of PDIA3 reduced the viability of LN229 cells, while KD of NEDD4L increased cell viability in an IKE dose‐dependent manner (Figure S3E,F, Supporting Information). Consistent with these results, depletion of PDIA3 sensitized cells to IKE‐induced cell death and lipid ROS accumulation, and depletion of NEDD4L suppressed IKE‐induced cell death and lipid ROS accumulation, for both LN229 (Figure 3E,F), and LN18 (Figure S3G,H, Supporting Information) cells, similar to the findings of a previous study.^[^ ^43^, ^44^ ^]^ Although KD of NEDD4L slightly hindered PDIA3 depletion‐mediated ferroptosis sensitivity, depletion of PDIA3 significantly eliminated the resistance to ferroptosis that mediated by NEDD4L depletion both in LN229 (Figure 3G,H) and LN18 cells (Figure 3I; Figure S3I, Supporting Information). Collectively, these data suggest that PDIA3 stability is potentially regulated by E3 ubiquitin ligase NEDD4L and is necessary for IKE‐induced ferroptosis.

### 2.4 NEDD4L Mediates K29‐Dependent Ubiquitination Degradation of PDIA3

**Figure 4:**
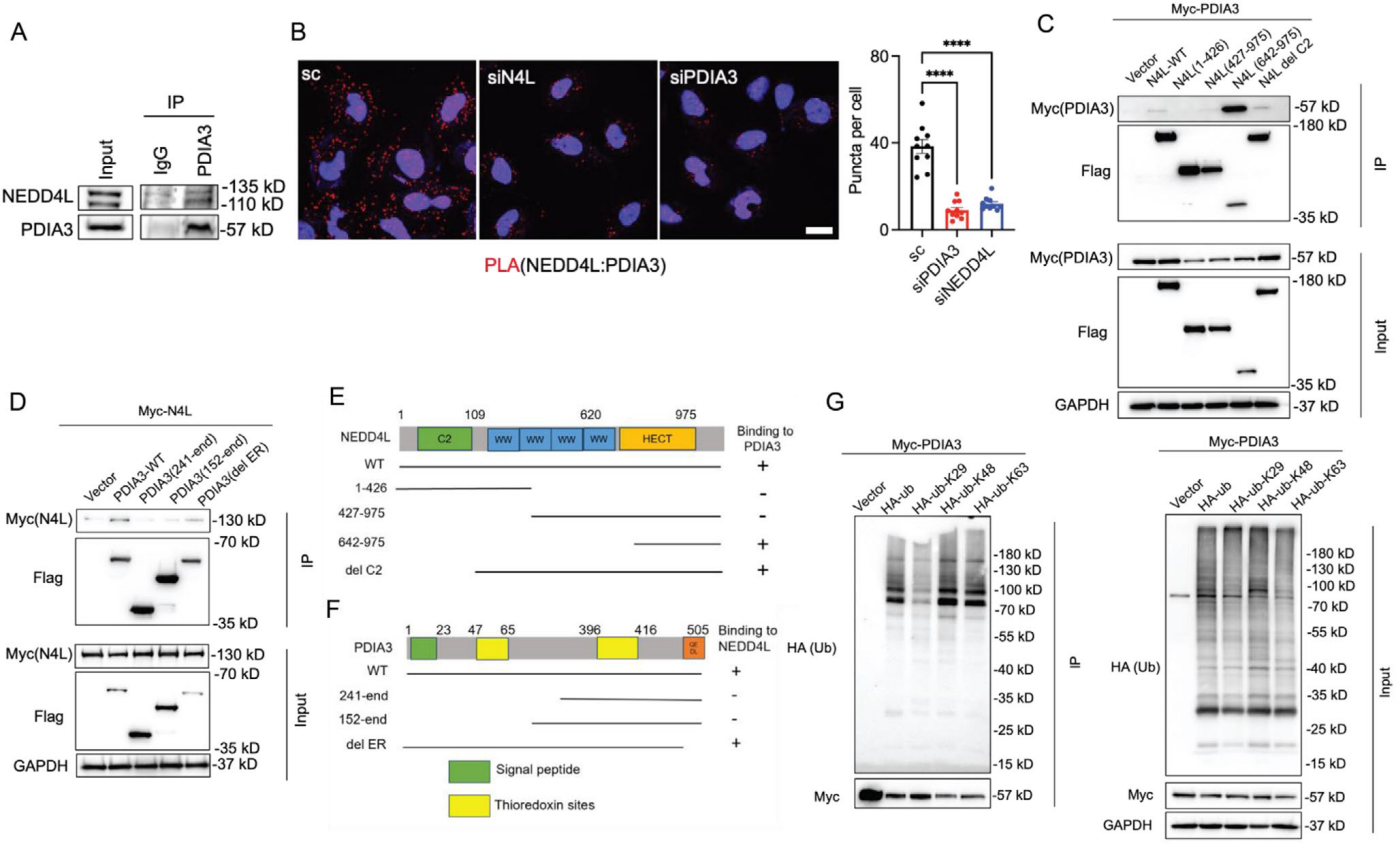
NEDD4L facilitates K29‐dependent ubiquitination degradation of PDIA3. A) Western blot was used to determine the protein expressions of NEDD4L and PDIA3 after endogenous immunoprecipitation was carried out in LN229 cells. B) Binding of PDIA3 and NEDD4L was detected by PLA assay after transfection of LN229 cells with the indicated siRNAs. LN229 cells, in which NEDD4L and PDIA3 were depleted by siRNA transfection, respectively, were seeded on coverslips and subjected to PLA using antibodies against NEDD4L and PDIA3. PLA signals (dots) in each cell were quantified. PLA signals (dots) in each cell were quantified. Two‐way ANOVA. ****p < 0.0001. Each data point corresponds to an image field that contains an average of 10 cells. *n* = 6–10 images for each condition as indicated. All images were collected from one experiment. Two independent experiments were performed, with similar results. Scale bar: 20 µm. C) Binding of truncated NEDD4L to PDIA3 was detected by co‐immunoprecipitation and western blotting. 293T cells were transfected with the indicated plasmids. D) Binding of truncated PDIA3 to NEDD4L was detected by co‐immunoprecipitation and western blotting. 293T cells were transfected with the indicated plasmids. E) Schematic diagram of the NEDD4L truncation. F) Schematic diagram of the PDIA3 truncation. G) Ubiquitination of PDIA3 was detected by Myc co‐immunoprecipitation, after 293T cells had been transfected with the indicated plasmids.

To determine how NEDD4L mediates ubiquitination degradation of PDIA3, we confirmed the interaction between NEDD4L and PDIA3 by endogenous immunoprecipitation using anti‐PDIA3 (**Figure** **4**A). We then conducted a proximity ligation assay (PLA). When expression of PDIA3 and NEDD4L was silenced by either of two different siRNAs, the PLA signals were eliminated (Figure 4B), indicating that the PLA signal is specific for the NEDD4L‐PDIA3 interaction. To further characterize the interaction between NEDD4L and PDIA3, we mapped the domains in each protein responsible for their interaction and found that the HECT domain of NEDD4L (Figure 4C,E) and the N‐terminal domain of PDIA3 (Figure 4D,F) were responsible for the NEDD4L–PDIA3 interaction. To further clarify the type of ubiquitination of PDIA3 mediated by NEDD4L, we constructed plasmids of HA‐Ubiquitin (Ub), HA‐Ub (K29R), HA‐Ub (K48R), and HA‐Ub (K63R), and co‐transfected them with myc‐PDIA3 into HEK293T cells; K29‐linked ubiquitination was significantly attenuated in these cells (Figure 4G). However, we further investigated whether the ubiquitination of PDIA3 is characterized by K29/K48 branched chains. The results indicated that PDIA3 was slightly polyubiquitinated at K48 under MG132 treatment, as demonstrated using a K48‐linkage specific antibody (Figure S4A, Supporting Information). This suggests that while K29‐linked polyubiquitination is the predominant type, K29/K48 branched chains may also be present in the polyubiquitination of PDIA3. These results suggest that NEDD4L specifically mediates a significant portion of K29‐linked degradation of PDIA3 through its interaction with PDIA3, thereby sensitizing SLC7A11‐dependent ferroptosis.

### 2.5 PDIA3 Prevents Ferroptosis Sensitivity by STAT3/LCN2 Axis

**Figure 5:**
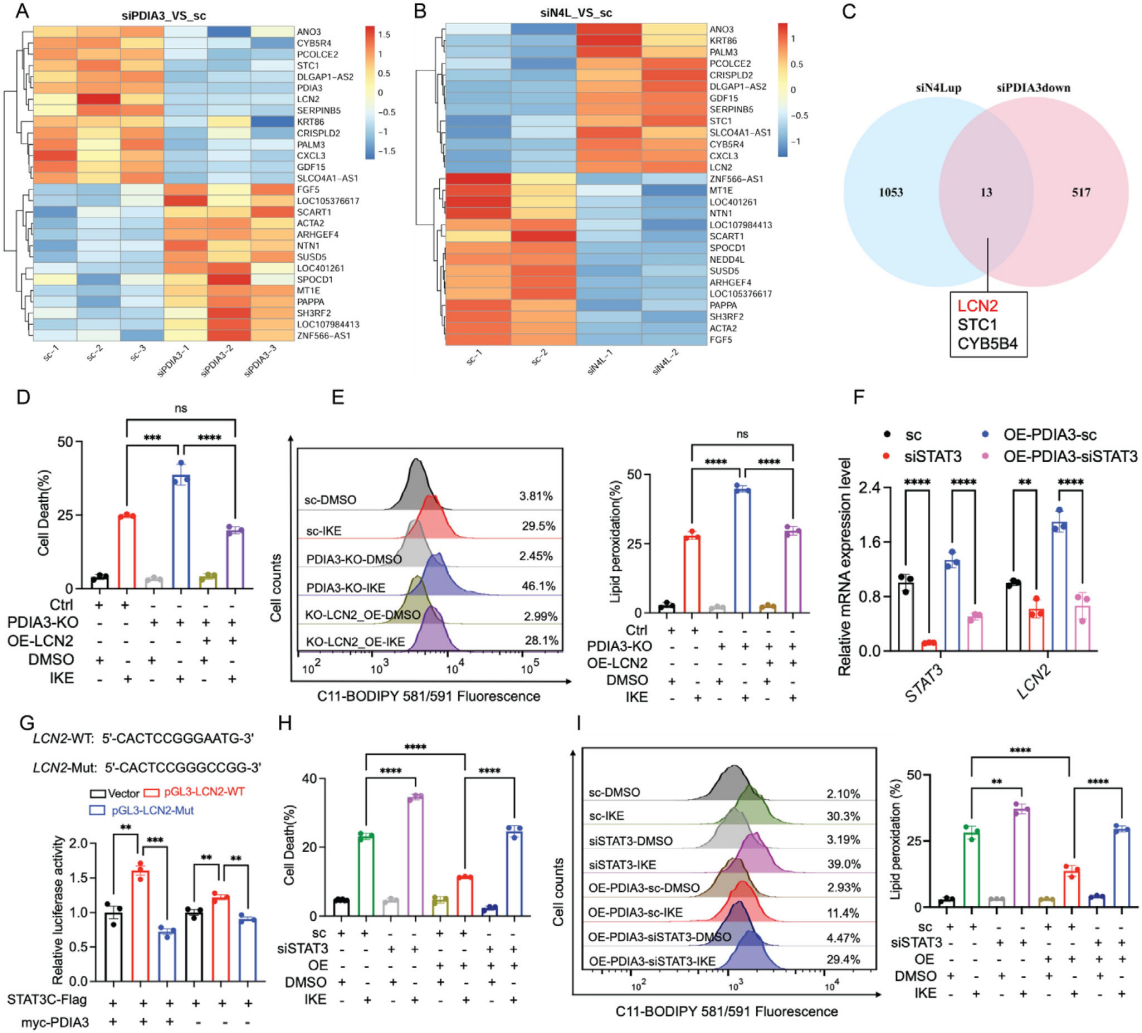
PDIA3 mediates ferroptosis sensitivity by STAT3/LCN2 axis. A, B) Heatmap representing differential expression of ferroptosis‐related genes in LN229 cells transiently transfected with siPDIA3 (A) or siNEDD4L (B) compared to the control group; gene activation (red) and inhibition (blue) are shown. C) Venn diagram of enriched genes shared between the set of upregulated ferroptosis‐related genes in LN229‐siNEDD4L cells and the set of downregulated ferroptosis‐related genes in LN229‐siPDIA3 cells. D) Histogram and flow cytometry results following C11‐BODIPY staining for detection of lipid peroxidation in cells subjected to 25 µm IKE treatment for 24 h (LN229 control, PDIA3 KO, and PDIA3‐KO cells transfected with the indicated plasmid). E) Detection and quantitative analysis of cell death via flow cytometry for cells subjected to 25 µm IKE treatment for 24 h (LN229 control cells, PDIA3 KO cells, and PDIA3‐KO overexpressing cells). F) Changes in mRNA expression levels of *LCN2* detected by qRT‐PCR in LN229 control and PDIA3‐overexpressing LN229 cells transfected with the indicated siRNAs. G) HEK293T cells transfected with pGL3 vector, LCN2 promoter or LCN2 promoter mutant‐Luc reporter, myc‐PDIA3, and STAT3C‐Flag as indicated were subjected to luciferase assay. The luciferase reading in each sample was normalized to that from cells transfected by the pGL3 vector‐Luc reporter alone in control cells. H). Detection and quantitative analysis of cell death via flow cytometry for cells subjected to 25 µm IKE treatment for 24 h (control and PDIA3‐overexpressing LN299 cells), transfected with the indicated siRNAs. I) Detection and quantitative analysis of lipid peroxidation in cells subjected to 25 µm IKE treatment (control cells and PDIA3‐overexpressing LN299 cells), transfected with the indicated siRNAs. The results are presented as mean ± standard deviation (SD). *n* = 3, **p < 0.01, ***p < 0.001, ****p < 0.0001. ns indicates no significance. OE, overexpression.

To further elucidate the mechanism underlying PDIA3‐mediated ferroptosis resistance, we conducted transcriptome profiling of LN229 cells transfected with NEDD4L and PDIA3 siRNAs. KD of the PDIA3 and NEDD4L genes significantly reshaped gene expression profiles of ferroptosis‐related genes (**Figure** **5**A,B). According to RNA sequencing analysis, 516 genes were upregulated, and 530 genes were downregulated in LN229‐siPDIA3 cells compared with control cells, whereas there were 1066 upregulated genes in LN229‐siNEDD4L cells and 1173 downregulated genes in LN229‐siNEDD4L cells (Figure S5A,B, Supporting Information). Three ferroptosis‐related genes, *LCN2*, *STC1* (stanniocalcin‐1), and *CYB5R4*, were found in the intersection between the upregulated genes in the siNEDD4L group and the downregulated genes in the siPDIA3 group (Figure 5C). To confirm that these candidate genes were regulated by PDIA3 and NEDD4L, we performed quantitative real‐time polymerase chain reaction (qRT‐PCR) analysis and found that *LCN2* expression was significantly decreased following PDIA3 depletion but increased following NEDD4L depletion (Figure S5C, Supporting Information). We further investigated the overexpression of PDIA3, which enhanced the mRNA expression of *LCN2* (Figure S5D, Supporting Information). However, neither the knockdown nor the overexpression of PDIA3 altered the expression of *SLC7A11*, the depletion of NEDD4L promoted the expression of *SLC7A11*, indicating PDIA3 may potentially regulate the function of SLC7A11 indirectly, but not its expression (Figure S5C,D, Supporting Information). These results identify LCN2 as a potential key gene regulated by both PDIA3 and NEDD4L, suggesting that expression of LCN2 has a vital role in PDIA3‐ and NEDD4L‐mediated ferroptosis.

To verify whether PDIA3 mediated ferroptosis resistance in a manner dependent on expression of LCN2, we constructed PDIA3 KO LN229 cells and overexpressed LCN2 in a subset of these cells. In comparison to the control group, PDIA3 KO enhanced IKE‐induced cell death. However, the overexpression of LCN2 in PDIA3 KO cells markedly reduced cell death compared to PDIA3 KO alone, resulting in no significant difference when compared to the control group (Figure 5D). Likewise, compared with control, KO of PDIA3 led to a significant increase in intracellular lipid peroxidation level, whereas exogenous overexpression of LCN2 could counteract this effect, restoring lipid peroxidation level to baseline (Figure 5E). These results suggested that overexpression of LCN2 could specifically reverse the increased sensitivity to IKE‐induced ferroptosis and accumulation of lipid peroxidation resulting from PDIA3 deficiency.

We next considered whether PDIA3 might regulate the expression of LCN2 through STAT3, as indicated by previous studies.^[^ ^45^, ^46^ ^]^ To determine whether STAT3 regulated LCN2 expression, we constructed an LN229 cell model with STAT3 KD using siRNA. qRT‐PCR and western blot results showed decreased *LCN2* mRNA and protein levels in this cell model (Figure 5F; Figure S5E, Supporting Information). Furthermore, PLA showed that PDIA3 interacted specifically with STAT3 in both cytoplasm and nucleus (Figure S5F, Supporting Information). To examine if PDIA3 regulates LCN2 expression through STAT3, we established LN229 cells that stably express PDIA3, with or without transfection with STAT3 siRNA. The qRT‐PCR results indicated that overexpression of PDIA3 significantly increased *LCN2* mRNA expression, whereas KD of STAT3 completely counteracted this effect, restoring LCN2 expression to baseline levels (Figure 5F). To investigate whether STAT3, with or without PDIA3, can directly promote the transcriptional expression of *LCN2* genes, we employed an LCN2 promoter reporter assay. As expected, ectopic expression of STAT3 alone significantly increased the activity of the LCN2 wild‐type promoter reporter, while it did not affect the LCN2 mutant in HEK293T cells (Figure 5G). Furthermore, the expression of PDIA3 further enhanced the reporter activity of the LCN2 wild‐type promoter mediated by STAT3 (Figure 5G). These results suggest that STAT3 is a crucial downstream factor through which PDIA3 regulates LCN2 expression. Subsequently, KD of STAT3 was found to enhance IKE‐induced cell death, whereas overexpression of PDIA3 reversed cell death under conditions of STAT3 deficiency (Figure 5H). We also found that STAT3 KD significantly increased lipid peroxidation levels, an effect that was counteracted by the overexpression of PDIA3 (Figure 5I). To further investigate whether PDIA3 regulates LCN2 expression through the nuclear translocation of STAT3, we examined the phosphorylation level of STAT3 in LN229 cells transfected with either siRNA or PDIA3 overexpression constructs. The results indicated that Y705 phosphorylation of STAT3 was not significantly affected by either the KD or OE of PDIA3 Figure S5G,H, Supporting Information). However, the overexpression of PDIA3 did enhance S727 phosphorylation of STAT3 and the total protein level of STAT3, notably, the KD of PDIA3 reversed this phenotype (Figure S5G,H, Supporting Information), indicating that PDIA3 promotes the expression and activation of STAT3 at S727. Furthermore, we observed that the ubiquitination of STAT3 was not influenced by either the depletion or overexpression of PDIA3 (Figure S5I, Supporting Information), suggesting that PDIA3 enhances the expression of STAT3 independently of its ubiquitination. Taken together, these results indicated that PDIA3 may maintain the ferroptosis‐resistant phenotype in GBM through the STAT3/LCN2 axis.

### 2.6 PDIA3 Inhibition Enhances Sensitivity of Ferroptosis to Improve GBM Therapy in Xenograft Tumors

**Figure 6:**
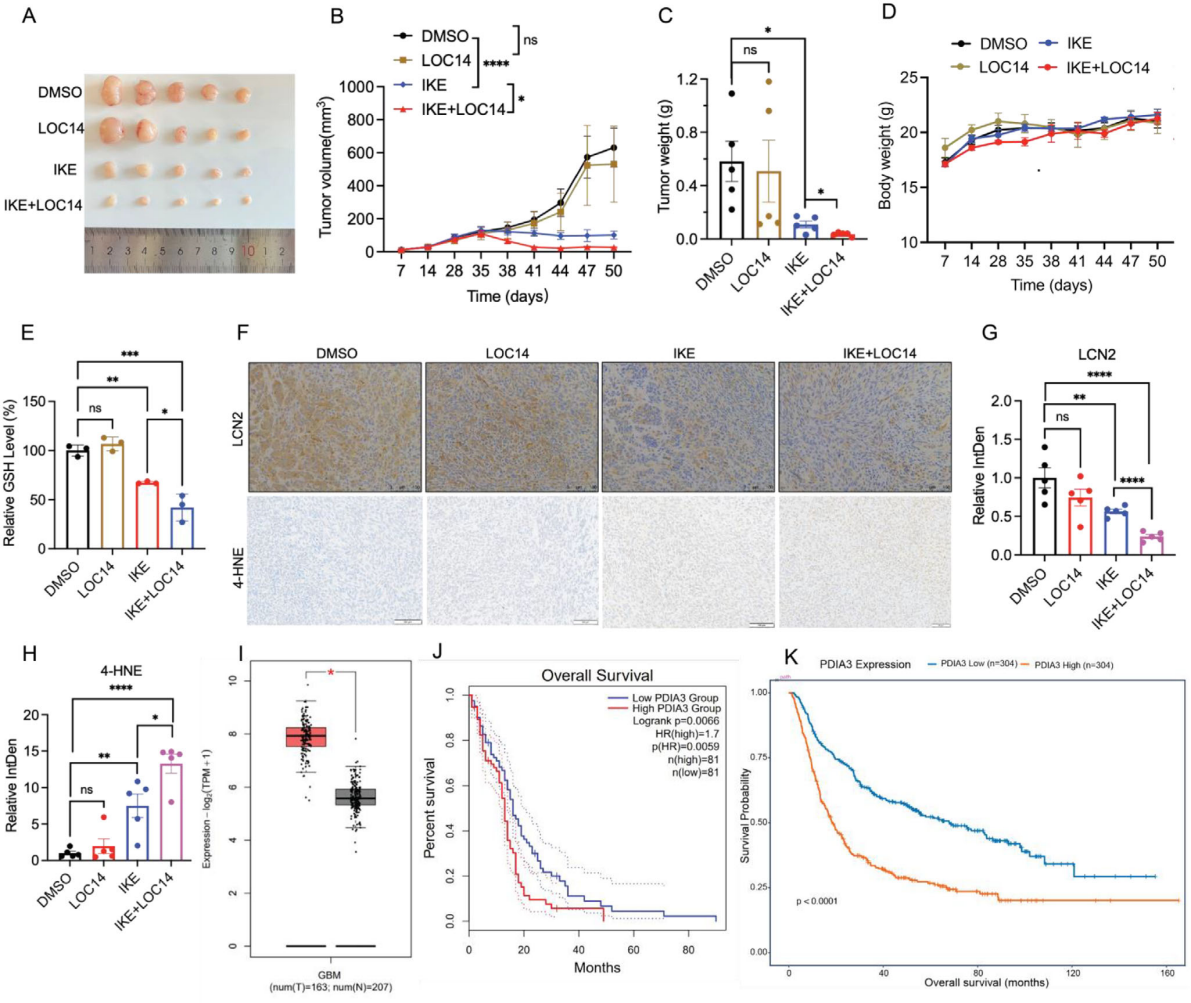
PDIA3 inhibition enhances sensitivity to ferroptosis to improve GBM therapy in xenograft tumors. A) Tumors were collected when the DMSO control group in B) reached the endpoint (day 50 after injection). C) Tumor weight chart for mice in the indicated groups. D) Body weights of mice in the indicated groups. E) Detection of GSH levels in mouse tumor tissues. F) 4‐HNE and LCN2 staining results of tumor tissue sections from mice in each group. Scale bar: 100 µm. G,H) Quantitative analysis of LCN2 (G) and 4‐HNE (H) of tumor tissue sections from mice in each group using Image J software. I) Expression levels of PDIA3 in normal tissues and GBM tissues from TCGA database. J,K) Overall survival curve for patients in the PDIA3 high and low expression groups using data from TCGA (J) and CGGA (K) dataset. The results are presented as mean ± standard deviation (SD), *p < 0.05, **p < 0.01, ***p < 0.001, ****p < 0.0001, and ns indicating no significance.

To further confirm that inhibition of PDIA3 could enhance GBM therapy in combination with ferroptosis induction, we established a subcutaneous tumor model of GBM in nude mice. After subcutaneous injection of LN229 cells for 35 days, drug treatment was initiated. Tumor volume was assessed every 2 days, and the mice were euthanized after 50 days of treatment. And tumors were weighed and photographed. Tumor volumes (**Figure** **6**A,B) and weights (Figure 6C) in the IKE/LOC14 combination treatment group were much lower than those in either the control or the IKE‐only group, indicating that the combination treatment was more effective than IKE treatment alone. Both treatment groups showed gradual increases in tumor volumes (Figure 6B,C), but body weights of tumor‐bearing mice showed no significant change as a result of drug treatment (Figure 6D), indicating the safety of the dosing regimen. GSH levels in tumor tissues in the combination treatment group were significantly lower than those in the IKE‐only group (Figure 6E). Consistent with the findings of our in vitro cell experiments, 4‐HNE immunochemical staining of tumor tissues revealed higher lipid peroxidation levels in the combination treatment group compared to the monotherapy group (Figure 6F,H). In addition, LCN2 staining showed markedly reduced expression of LCN2 in the combination treatment group compared to the monotherapy group (Figure 6F,G). We also investigated whether PDIA3 was associated with the clinical prognosis of GBM patients; based on integrated analysis of data from TCGA and the Genotype‐Tissue Expression database, we found that PDIA3 showed significant upregulation in tumor tissues from GBM patients (*n* = 163) compared to normal brain tissue (*n *= 207) (Figure 6I). Moreover, we observed that higher PDIA3 expression was correlated with shorter survival of patients either in the TCGA GBM (Figure 6J) or Chinese Glioma Genome Atlas (CGGA) cohort (Figure 6K). We further investigated subgroup survival analyses, which stratified prognostic outcomes among distinct patient groups based on multiple established factors. The results indicated that higher PDIA3 expression was significantly associated with poor prognosis, correlating with recurrence, IDH mutation status, patient age, and WHO grading. However, the aforementioned prognostic factors did not influence the prognostic value of PDIA3 (Figure S6A–D, Supporting Information). Univariate analysis demonstrated a strong correlation between PDIA3 expression and IDH mutation status, tumor grade, and overall survival (OS), while no significant association was observed between patient age groups and OS (Figure S6E, Supporting Information). These findings suggest that PDIA3 serves as an important prognostic biomarker for patients with various types of gliomas, significantly correlating with OS. These results suggest that the combined use of a PDIA3 inhibitor and IKE is more effective as a treatment for GBM than monotherapy, indicating a potential novel therapeutic approach for clinical treatment of GBM.

### 2.7 Synthesis and Characterization of TDN‐IKE

**Figure 7:**
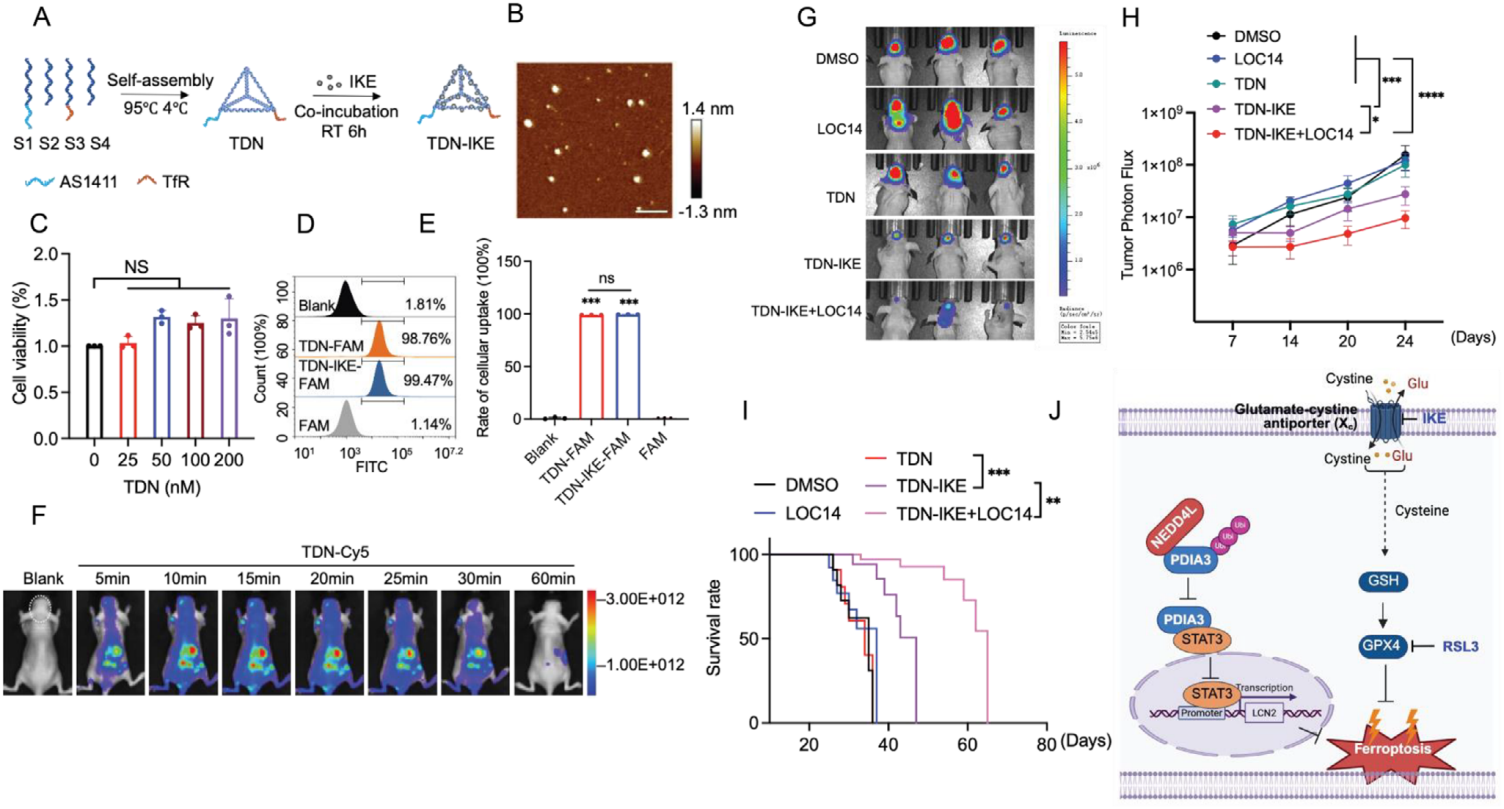
Combined use of TDN‐IKE and LOC14 exerts an antitumor synergistic effect by penetrating BBB. A) Schematic diagram of the synthesis and morphology of TDN materials, AS1411: DNA aptamers of nucleolin; TfR: transferrin receptor. B) AFM image of TDN displayed by atomic force microscopy. C) Histogram showing effects of 24 h treatment with different concentrations of TDN on LN229 cells. D,E) The proportion of free FAM, fluorescently labeled TDN, and TDN‐IKE uptake by LN229 cells (4 h) via flow cytometry. Blank, unstained cells. F) In vivo imaging of BBB penetration at different times after TDN injection in mice. G) Bioluminescence images of mice in the experiment shown in H) at day 24 after injection of LN229 cells. DMSO, control group; LOC4, LOC14 treatment group; TDN, TDN control group; TDN‐IKE, IKE‐loaded TDN group; TDN‐IKE+LOC14: IKE‐loaded TDN and LOC14 combined treatment group. I) Kaplan–Meier survival curves for LN229 tumor‐bearing mice in each group, *n *= 5. J) A proposed model showing how PDIA3 is involved in regulating IKE‐induced ferroptosis in GBM. The results are presented as mean ± standard deviation (SD), *p < 0.05, **p < 0.01, ***p < 0.001, ****p < 0.0001, and ns indicating no significance.

As IKE has poor BBB permeability, we used a TDN as a delivery material to help IKE penetrate the tumor site. This comprised a novel DNA nanomaterial with a tetrahedral spatial structure and excellent permeability into brain tissue.^[^ ^47^, ^48^ ^]^ As shown in **Figure** **7**A, the TDN‐IKE was synthesized by a two‐step process. First, four predesigned single‐stranded DNA sequences were self‐assembled based on base complementarity to form TDN (Table S4, Supporting Information). Notably, AS1411, a DNA aptamer that specifically targets nucleolin and is highly expressed on the surface of glioma cells,^[^ ^49^, ^50^ ^]^ was appended to the 3′ end of S1 to improve the tumor targeting efficiency. A transferrin receptor (TfR) aptamer, which has shown efficient penetration of the BBB,^[^ ^51^ ^]^ was added to the 3′ end of S3. Subsequently, IKE was loaded into TDN by co‐incubation at room temperature for 6 h. Agarose gel electrophoresis showed successful generation of TDN (Figure S7A, Supporting Information). The morphology of TDN was characterized by atomic force microscopy (AFM) (Figure 7B). To examine the cytocompatibility of TDN, its effects on the growth of LN229 cells were determined using a CCK‐8 assay. After 24 h of culture, TDN showed little effect on cell viability (Figure 7C). Furthermore, after successful loading of IKE into the TDN (resulting in TDN‐IKE), we calculated the encapsulation efficiency according to the standard high‐performance liquid chromatography (HPLC) curve of IKE (Figure S7B, Supporting Information), and the particles with the highest encapsulation rate (53.84%) were used for subsequent experimental procedures (Figure S7C, Supporting Information). AFM images of TDN‐IKE showed that the IKE loading did not change the geometric structure of TDN (Figure S7D, Supporting Information). Moreover, both TDN and TDN‐IKE showed negative zeta potentials of about −20 mV (Figure S7E, Supporting Information). The particle diameters of TDN and TDN‐IKE were 18.8 nm and 19 nm, respectively, according to dynamic light scattering (DLS, Figure S7F, Supporting Information), and transmission electron microscopy (TEM) images revealed that both TDN and TDN‐IKE were ≈10 nm in size (Figure S7G, Supporting Information). In addition, the cellular uptake of TDN and TDN‐IKE was assessed using flow cytometry and confocal microscopy imaging, with the FAM fluorescent group was modified onto S4 for TDN tracing (Figure 7D,E; Figure S7H, Supporting Information). The results showed that TDN and TDN‐IKE were readily and effectively delivered into GBM cells. We then incubated TDN and TDN‐IKE with 5% fetal bovine serum (FBS) for 48 h to assess their serum stability (Figure S7I, Supporting Information). The results showed TDN‐IKE maintained better structural stability than TDN, with little degradation after 24 h of incubation.

To investigate the BBB penetration of TDN and TDN‐IKE, we used an in vivo imaging system. This demonstrated their distribution across multiple organs, including the brain (Figure 7F). Intriguingly, 5 min after TDN and TDN‐IKE had been injected into the tail vein, few TDN particles had accumulated in the brain region, although large amounts were distributed in the liver. To investigate the targeted effects of TDN‐mediated IKE in brain tumor tissues, we performed liquid chromatography mass spectrometry analysis to detect IKE in brain GBM tissues and healthy brain tissues 2 h of tail vein injection of TDN‐IKE. We observed 4–5‐fold higher concentrations of IKE in tumor tissues compared to healthy tissues, indicating that TDN‐mediated IKE specifically accumulates in GBM tumor tissues (Figure S7J, Supporting Information). Hence, we concluded that TDN/TDN‐IKE could penetrate the BBB and rapidly gather in the brain area. Based on these results, TDN was used as a nanocarrier for brain delivery of IKE in subsequent experiments.

### 2.8 Combined Use of TDN‐IKE and LOC14 Exerts an Antitumor Synergistic Effect by Penetrating the BBB

Given the specificity of the glioma microenvironment, this study employed a blood–brain barrier‐penetrating nanocarrier (TDN) loaded with the ferroptosis inducer IKE (TDN‐IKE) to overcome the obstacles associated with drug delivery.

On day 7 after tumor inoculation, we conducted in vivo imaging of fluorescence in our tumor‐bearing mouse. This was followed by alternating group interventions starting from day 9 (DMSO control group, LOC14 group, TDN empty vector group, TDN‐IKE group, and TDN‐IKE+LOC14 combined treatment group). In vivo imaging showed a significant reduction in tumor fluorescence intensity in the TDN‐IKE group compared with the control group (Figure 7G,H), with greater inhibition of tumor growth compared to the TDN‐IKE monotherapy group, suggesting a synergistic antitumor effect of the PDIA3 inhibitor and the ferroptosis inducer (Figure 7H). After treatment, physiological indicators of the mice were continuously monitored, and euthanasia was performed when preset endpoints were reached. The median survival time was significantly longer in the TDN‐IKE group compared to the control group (Figure 7I), and it was further extended in the combined treatment group.

### 2.9 Biosafety Evaluation of TDN‐IKE

Encouraged by the effective therapeutic results achieved by TDN‐IKE in a murine GBM model, we then determined the biosafety of TDN‐IKE in vitro and in vivo. In vitro red blood cell (RBC)‐induced hemolysis is a reliable method for assessing the safety of biomaterials. In this study, we evaluated the hemolytic activity induced by TDN at various concentrations. The data indicated that TDN did not induce hemolysis at concentrations up to 1 µm, suggesting that TDN exhibits good blood compatibility (Figure S8A, Supporting Information). And we also measured the endotoxin level of drugs in the different treatment groups using limulus amebocyte lysate (LAL) assays, and the result showed LAL levels were within the normal range and nontoxic (Figure S8B, Supporting Information).

Notably, complement activation and serum biochemistry assays were conducted on day 24 following multiple intravenous administrations of TDN‐IKE. As expected, C3 protein levels after treatment of TDN‐IKE were in the normal range and consistent with PBS group of healthy mice (Figure S8C, Supporting Information). The liver and kidney function parameters, including alanine aminotransferase (ALT), aspartate aminotransferase (AST), creatinine (Cr), and blood urea nitrogen (BUN), remained within the normal range and showed no significant difference compared to the PBS group (Figure S8D–G, Supporting Information). Furthermore, when compared to healthy mice, the body weights of mice treated with TDN‐IKE exhibited negligible differences (Figure S8H, Supporting Information). These results indicate that TDN‐IKE did not cause any adverse side effects. Hematoxylin and eosin staining also showed no significant pathological damage to vital organs, confirming the safety of the dosing regimen (Figure S8I, Supporting Information). Taken together, these findings provided potential preclinical evidence for PDIA3 as a promising target in the treatment of GBM or other malignant tumors.

## 3 Discussion

In this study, we identified PDIA3 as a crucial PDI family member that influences the susceptibility of GBM cells to ferroptosis triggered by IKE and cystine starvation. Inhibition or KD of PDIA3 increased the sensitivity of GBM cells to ferroptosis, whereas its overexpression inhibited ferroptosis. We also identified NEDD4L as a novel E3 ubiquitin ligase of PDIA3 that directly interacts with it to mediate its ubiquitination; this process also increased the sensitivity of GBM cells to IKE‐induced ferroptosis. NEDD4L facilitates K29‐dependent ubiquitin degradation of PDIA3 through the interaction between its C‐terminal HECT domain and the N‐terminal of PDIA3. In addition, PDIA3 modulates ferroptosis susceptibility through expression of LCN2, which in turn is regulated by STAT3. We also developed a drug delivery system using a TDN encapsulating IKE (TDN‐IKE) to penetrate the BBB. The combination of LOC14 and TDN‐IKE exhibited a synergistic antitumor effect in vivo, providing a foundation for future development of novel GBM therapies based on the induction of ferroptosis (Figure 7J).

Multiple studies have reported that PDI family members, including PDI (P4HB), PDIA4, and TXNDC12, play important parts in the mediation of chemically induced ferroptosis in breast, liver cancer, and neuronal disorders via accumulation of cellular lipid ROS and decreases in GSH content.^[^ ^34^, ^35^, ^37^, ^52^ ^]^ For instance, KD of PDIA1 enhances the sensitivity of IKE‐induced ferroptosis in breast cancer,^[^ ^52^ ^]^ and gallium metal can interfere with the GSH system via inhibition of PDIs, in turn downregulating SLC7A11, which controls lipid peroxide clearance and cystine uptake, and ultimately results in ER stress that leads to ferroptosis in breast cancer cells.^[^ ^36^ ^]^ In renal cell carcinoma, PDIA4 promotes ferroptosis resistance via induction of ATF4/SLC7A11.^[^ ^37^ ^]^ TXNDC12, which is also a member of the PDI family, mediates erastin‐induced ferroptosis by directly interacting with SLC7A11 to regulate lipid peroxidation, Fe^2+^ levels, and GSH content in glioma,^[^ ^34^ ^]^ as well as inhibiting lipid peroxidation in a GPX4‐independent manner without affecting iron accumulation; it is thus an ATF4‐dependent target gene in leukemia.^[^ ^35^ ^]^ In the present study, we identified a novel role for PDIA3 in mediating ferroptosis resistance in GBM cells. Although depletion of ERP29 notably facilitated IKE‐induced ferroptosis, it did not enhance PDIA3‐ or LOC14‐mediated susceptibility to ferroptosis. Contrary to expectations, PDI inhibitor LOC14 did not facilitate IKE‐mediated ferroptosis sensitivity but slightly decreased IKE‐induced ferroptosis. These results indicate potentially different mechanisms of PDIA3 and ERP29 in IKE‐mediated ferroptosis in GBM cells, with LOC14 unable to cross‐react across inhibition of ERP29. However, further investigation will be required to explore the mechanism involving ERP29. Furthermore, the depletion of PDIA3 significantly reduces the IKE‐induced GSH levels in GBM cells. Under IKE treatment, the ubiquitination of PDIA3, mediated by NEDD4L, induces prolonged ER stress, which may disrupt its function. This disruption results in the oxidation of GSH, consequently promoting ferroptotic cell death. Silencing of PDIA3 significantly increased susceptibility to ferroptosis, while PDIA3 inhibition markedly enhanced IKE‐induced ferroptosis in GBM cells in an ATF4‐independent manner (Figure S5J, Supporting Information), accompanied by notable increases in lipid peroxidation levels. Therefore, further investigation is warranted to explore the mechanism by which PDIA3 depletion diminishes IKE‐induced GSH loss.

NEDD4L contributes to ferroptosis by facilitating SLC7A11 ubiquitination and tumor growth inhibition in esophageal squamous cell carcinoma(ESCC)^[^ ^43^ ^]^ and non‐small cell lung cancer (NSCLC).^[^ ^53^ ^]^ Similarly, drug‐induced lactate has been shown to enhance the production of mitochondrial ROS and activate the p38‐SGK1 pathway by reducing the interaction of NEDD4L with GPX4, leading to ubiquitination and degradation of GPX4 in non‐small cell lung cancer (NSCLC).^[^ ^44^ ^]^ By contrast, NEDD4L deficiency in intestinal epithelial cells has been reported to inhibit expression of the key ferroptosis regulator glutathione peroxidase 4 (GPX4) by modulating ubiquitinoylation of solute carrier family 3 member 2 (SLC3A2) without affecting its gene expression, ultimately promoting dextran sulfate sodium (DSS)‐induced IEC ferroptosis of intestinal epithelial cells in vitro and in vivo.^[^ ^54^ ^]^ Intratumoral Brevibacillus parabrevis enhances antitumor immunity by inhibiting ferroptosis of natural killer cells via NEDD4L‐induced ubiquitination of iron transporters SLC39A14, SLC39A8, and STEAP3 in hepatocellular carcinoma.^[^ ^55^ ^]^ This accumulating evidence indicates that NEDD4L mediates ferroptosis by facilitating ubiquitination and degradation of multiple substrates in different contexts. Here, our results showed that PDIA3 protein was unstable during IKE‐induced cell ferroptosis, and that NEDD4L facilitated the degradation of PDIA3 through its HECT domain interaction. This suggests that NEDD4L‐mediated ubiquitination of PDIA3 increases the vulnerability of GBM to ferroptosis. However, the multiple potential ubiquitination sites were predicted using the software DeepMVP (Figure S4B, Supporting Information), and further investigation is needed to identify the key site of NEDD4L‐mediated ubiquitination of PDIA3 in the future.

Mechanistically, it is noteworthy that the inhibition of STAT3 activity triggers ferroptosis in several diseases through lipid peroxidation and the accumulation of Fe^2+^, effects that are mediated by the regulation of GPX4, SLC7A11, and FTH1 expression.^[^ ^56^, ^57^, ^58^ ^]^ Activation of STAT3 promotes expression of the iron cytokine Lipocalin 2 (LCN2), an antioxidant‐related protein that is upregulated in various cancers, thereby facilitating the progression of oral squamous cell carcinoma (OSCC) and non‐small cell lung cancer (NSCLC).^[^ ^59^, ^60^ ^]^ This suggests that LCN2 is a significant downstream target gene of STAT3. Recent studies have identified the major vault protein as an oncogenic RNA‐binding protein that mediates sorafenib resistance via suppression of ferroptosis through directly binding to the mRNA of iron‐sequestering cytokine *LCN2* and maintaining stability of LCN2 expression in hepatocellular carcinoma (HCC).^[^ ^61^ ^]^ LCN2 also mediates ferroptosis resistance in HCC through regulation by the LIFR−NF‐κB axis,^[^ ^62^ ^]^ and the NUPR1/LCN2 pathway blocks ferroptosis through reducing iron accumulation and subsequent oxidative stress in pancreatic cancers.^[^ ^63^ ^]^ Depletion of LCN2 via CRISPR/Cas9 KO effectively facilitates erastin‐mediated ferroptosis.^[^ ^64^, ^65^ ^]^ In the present study, the expression of SLC7A11 does not significantly change with either the knockdown or overexpression of PDIA3. However, the depletion of NEDD4L facilitates the expression of SLC7A11, suggesting that while PDIA3 may influence the functional activity of SLC7A11, it does not appear to directly affect its expression. It is possible that PDIA3 regulates the function of SLC7A11 indirectly. Nevertheless, the mechanism by which PDIA3 primarily acts on the cystine/glutathione axis, rather than on GPX4, remains to be clarified. Furthermore, PDIA3 KD substantially reduced LCN2 expression, whereas PDIA3 overexpression promoted LCN2 expression in a manner dependent on STAT3 transcription. Furthermore, LCN2 overexpression rescued ferroptosis induced by PDIA3 and STAT3 deficiency in GBM cells, suggesting that PDIA3 prevents ferroptosis through a mechanism dependent on expression of LCN2. These findings demonstrate that PDIA3 is a key regulator of ferroptosis susceptibility in GBM and provide preliminary insight into the molecular mechanism by which PDIA3 influences ferroptosis sensitivity through STAT3/LCN2 expression. The PDIA3‐STAT3/LCN2 axis warrants further investigation, in particular, with respect to the molecular mechanism of its regulation in the nucleus. Future work should aim to establish the relationships between PDIA3 expression and biological processes, including iron metabolism and ferroptosis.

## 4 Experimental Section

### Cell Culture and Treatment

Human GBM cell lines, comprising LN229 cells (CL‐0578) and LN18 cells (CL‐0846), were purchased from Procell Life Science & Technology (Wuhan, China). All cells were grown at 37 °C, 5% CO2, in Dulbecco's Modified Eagle Medium (Gibco, 11965118) complemented with 10% FBS (Vazyme, F101‐01) and 1% penicillin–streptomycin (Gibco, 15140‐122). All cell lines were confirmed as Mycoplasma negative before experiments.

### Animals Models

Five‐ to eight‐week‐old female nude mice (BALB/cNj‐Foxn1nu/Gpt, D000521) were purchased from GemPharmatech (Jiangsu, China) and acclimated for 7 days in a specific‐pathogen‐free facility at 22 °C and 40–50% humidity, with a 12 h/12 h light–dark cycle before experiments. Animals were randomly allocated into different groups. All animal experiments were conformed to the ethical guidelines and protocols (W202504150528) approved by the Animal Care and Use Committee of Shandong First Medical University (Shandong Academy of Medical Sciences), and all protocols comply with the ethical guidelines for the care and use of non‐human animals in scientific research.

### Subcutaneous Xenograft Tumor Model

A total of 8.0 × 10^6^ LN229 cells were suspended in 100 µL of phosphate‐buffered saline (PBS) and injected subcutaneously into female nude mice. After tumor formation, 200 µL IKE (40 mg/kg) and/or 200 µL LOC14 (20 mg kg^−1^), dissolved in 5% DMSO or a vehicle, were administered intraperitoneally every other day, starting on day 35. Seven days later, the tumor volumes were measured, and the mice were sacrificed after 50 days. Tumors were weighted, photographed, and processed for further histological analysis and GSH assays. Tumor volume was calculated as follows: V (volume) = (length × width^2^)/2.

### Orthotopic Xenograft Tumor Model

LN229 cells were transduced with a retroviral vector that expressed firefly luciferase. For each mouse, 3.5 × 10^5^ cells were injected into the right hemisphere at coordinates (+1, +2, −3). Bioluminescence imaging was used to track the development of tumors in mouse brains. Briefly, the mice were anesthetized with isoflurane, then injected intraperitoneally with 0.75 g of D‐luciferin potassium salt (Beyotime, ST196) dissolved in 50 mL D‐PBS (Beyotime, C0221D), with a total injection volume of 200 µL. The mice were placed in an IVIS imaging chamber (IVIS Spectrum CT, PerkinElmer), 10 min later, and photons released from the brain region were measured using the Living Image software, with luciferase activity was expressed as photons emitted per second. IKE was incorporated in the Tetrahedral DNA Nanostructure (TDN), which is also referred to as TDN‐IKE. Starting 7 days later, 100 µL TDN‐IKE (20 mg kg^−1^) and 100 µL TDN (1 µm) were intravenously injected every other day. A total of 50 µL LOC14 (10 mg kg^−1^) was injected intraperitoneally into mice every other day.

### RNA Extraction and qRT‐PCR

A total of 5.0 × 10^5^ cells were seeded in a single well of a six‐well plate 24 h prior to the experiments. RNA was extracted using an RNA Rapid Extraction Kit (Spark Jade, AC0205‐B), and qRT‐PCR was performed with a BIOER(FQD‐96C) and ChamQ SYBR Color qPCR Master Mix (Q411‐02). The primers used are listed in Table S1 (Supporting Information).

### siRNA Transfection

The siRNAs for human P4HB, PDIA3, and NEDD4L were purchased from RiboBio (Guangzhou, China). Human PDIA2, PDIA4, PDIA5, PDIA6, ERp29, STAT3, and ATF4 siRNAs were purchased from Keyybio (Shandong, China). The sequences are listed in Table S2 (Supporting Information). Transfections with plasmids and siRNAs were performed with jetPRIME Transfection Reagent (Polyplus, Strasbourg, France, 101000046) according to the manufacturer's instructions. Cells were collected 48 h after siRNA delivery for subsequent assays.

### Co‐Immunoprecipitation (Co‐IP) Assay

For immunoprecipitation of endogenous protein, first, 3.0 × 10^6^ LN229 cells were seeded in a 10 cm plate overnight. Cells were then lysed in RIPA buffer (50 mm Tris, pH 7.5, 150 mm NaCl, 4 mm EDTA, 1 mm EGTA, 1%Triton X‐100, 0.5% sodium deoxycholate, 0.1% sodium dodecyl sulfate (SDS), 10% glycerol, 1× phosphatase inhibitor, and 1× proteinase inhibitor) or RIPA buffer without SDS as indicated. Cell extracts were cleared by centrifugation, incubated with anti‐PDIA3 (Proteintech, 66423), anti‐NEDD4L (Cell Signaling Technology, 4013), or mouse IgG (Sigma–Aldrich, I5381) antibodies at 4 °C overnight, then incubated with protein A/G agarose beads (Thermo Scientific, 20423) for a further 2 h. The precipitates were washed with RIPA buffer three times. Bound proteins were dissociated in 20 µL of SDS sample buffer (25 mm Tris, pH 6.8, 4% SDS, 5% glycerol, and bromophenol blue) before being separated by SDS polyacrylamide gel electrophoresis (Sevenbio, Beijing, China) and subjected to standard immunoblotting.

For immunoprecipitation of Flag‐tagged proteins, 293T cells were transfected with the indicated plasmids overnight, lysed, and then incubated with benzonase nuclease in RIPA buffer as described above. The precipitates were washed three times with IP2 buffer (50 mm Tris, pH 7.5, 150 mm NaCl, 4 mm EDTA, 1 mm EGTA, 0.5% NP‐40, 10% glycerol, 1× phosphatase inhibitor and 1× proteinase inhibitor), and eluted with 3x Flag Peptide (Sigma #F329020) dissolved in TBS (10 mm Tris–HCl, 150 mm NaCl, pH 7.4). The proteins were then separated by SDS‐polyacrylamide gel electrophoresis (Sevenbio, Beijing, China) and subjected to standard immunoblotting.

### Western Blot

A total of 1.0 × 10^6^ cells were seeded 24 h before experiments, and then lysed in SDS‐lysis buffer consisting of 10 mm Tris (pH 7.5), 1% SDS, 50 mm NaF, and 1 mm NaVO4. The BCA protein assay kit (Thermo Scientific, 23227) was used to measure protein concentrations. The 20–40 µg protein from each sample was loaded in SDS sample buffer as described above and separated in running buffer. The protein was transferred to a nitrocellulose blotting membrane (Millipore, China, HATF00010), and subsequently blocked in 5% skim milk/TBST (0.1% Tween, 10 mm Tris at pH 7.6, 100 mm NaCl) for 1 h at room temperature. The membranes were incubated with primary antibodies (1:1000) overnight at 4 °C and then with the secondary antibody (1:2000) at room temperature for 1 h. SuperSignal West Pico PLUS (Thermo Scientific, 34580) was used to detect proteins, with Tanon 4800CE Multi Chemiluminescence imager (Tanon Science & Technology Co., Shanghai, China) was used in the study. The antibodies used are listed in Table S3 (Supporting Information).

### Cell Viability

A Cell Counting Kit‐8 (CCK‐8) assay (Spark Jade, CT0001) was used to measure the viability of cells. Briefly, cells were seeded into 96‐well plates with 100 µL medium per well overnight. Then, cells were treated with the indicated compounds, and 100 µL fresh medium containing 10 µL CCK‐8 solution was added to each well, followed by incubation for 2–4 h in the dark. Finally, cells were incubated at 37 °C with 5% CO_2_ for 2 h, and the absorbance at 450 nm was measured with a Molecular Devices Reader (Thermo Scientific, USA).

### GSH Level Measurement

Cells (4.0 × 10^5^) were seeded 24 h before experiments; then, they were collected, and their GSH contents were measured using a GSH Assay Kit (Nanjing Jiancheng, Jiangsu, China) in line with the manufacturer's instructions. The GSH contents were finally normalized to the corresponding total protein concentrations.

### Immunofluorescence Assay

Briefly, LN229 cells were seeded on a sterilized glass slide and fixed with 4% paraformaldehyde in PBS for 20 min and incubated in permeabilization buffer (PDT:0.3% sodium deoxycholate, 0.3% Triton X‐100 in PBS) for 30 min on ice. Samples were then blocked with 5% bovine serum albumin (BSA)/PBS at 4 °C for 1 h and followed by incubating overnight at 4 °C with primary antibodies diluted in 2.5% BSA/0.05% Triton X‐100/PBS. After washing with PBS buffer, fluorescent secondary antibodies (Boster Biological Technology, Wuhan, China) were added to the dishes, followed by incubation for 1 h at room temperature. Nuclei were stained with DAPI (Boster Biological Technology, Wuhan, China, AR1177), and the cells were photographed under a fluorescence confocal microscope (SpinSR10, Olympus, Japan). PLA was performed using an Affinity Purified Donkey Kit (Sigma, DUO92004/DUO92002). Cells were then prepared as described above for immunofluorescence staining and incubated overnight at 4 °C with primary antibodies, before undergoing further incubation with a mixture of the MINUS and PLUS PLA probes for 1 h at 37 °C. Hybridized probes were ligated using Ligation–Ligase solution for 30 min at 37 °C and then amplified with Amplification‐Polymerase solution for 100 min at 37 °C. Finally, cells were mounted with Duolink II Mounting Medium containing DAPI and imaged under a fluorescence confocal microscope (SpinSR10, Olympus, Japan).

### Luciferase Reporter Assay

Seed 293T cells in six‐well plates at 70% confluence, adding 2 mL of medium per well, and culture for 24 h. Transfer 1 µg of plasmid DNA into a 1.5 mL Eppendorf tube, then add 50 µL of serum‐free medium (consistent with the culture system, serum‐free and antibiotic‐free), and mix gently. In a separate 1.5 mL Eppendorf tube, add 2.5 µL of PEI Transfection Reagent and 50 µL of serum‐free medium (consistent with the culture system, serum‐free and antibiotic‐free), then mix gently. Combine the contents of both tubes and incubate at room temperature for 30 min. Subsequently, add the mixture to the six‐well plate and mix thoroughly. After an additional 24 h, lyse the cells and measure the luciferase signal using a luciferase assay kit (Vazyme, DL101‐01). Normalize the firefly luciferase luminescence signal of each sample to the Renilla luciferase signal. The specific binding site of STAT3 on the LCN2 promoter is located at −170 bp, with the specific sequence 5′‐CACTCCGGGAATG‐3′. This sequence was mutated to 5′‐CACTCCGGGCCGG‐3′ through site‐directed mutagenesis.

### Flow Cytometry

GBM cells were cultured with the drug treatment for 24 h. After washing twice with PBS and staining with 10 µm BODIPY 581/591 C11 (Thermo Scientific, D3861) or propidium iodide (MCE, HY‐D0815), cells were resuspended in 500 µL of PBS with 1% FBS and subjected to flow cytometry on a FongCyte Series 3‐Laser 14‐Color (Challen Biotechnology Co., Ltd, Beijing). The results were processed and visualized with FlowJo v. 10.8.1.

### Fabrication of TDN and TDN‐IKE

Raw DNA chains were synthesized by Sangon Biotech (Shanghai, China). For the fabrication of TDN, four single‐stranded DNA molecules (ssDNAs, Table S4, Supporting Information) (Sangon, Shanghai, China) were mixed in equimolar quantities, dissolved in TM buffer (Tris‐HCl and MgCl2, pH 8.0), heated at 95 °C for 10 min, and then cooled at 4 °C for 20 min.^[^ ^66^ ^]^ TDN‐IKE was prepared by incubation of different concentrations of IKE solution (50, 100, 150, and 200 µm) with TDNs (250 nm) at room temperature overnight, and then ultrafiltrated with an Amicon ultrafiltration device (30 K) to remove the unattached IKE. The IKE encapsulation efficiency was determined using the high‐performance liquid chromatograph (HPLC) and calculated by the following formula:

(1) $$\begin{array}{ccc} \mathrm{encapsulation}\;\mathrm{efficiency} & = & \left(\mathrm{loading}\;\mathrm{concentration}\;\mathrm{of}\;\mathrm{IKE}\;\mathrm{into}\;\mathrm{TDN}\right)/\\ & & \times \left(\mathrm{initial}\;\mathrm{concentration}\;\mathrm{of}\;\mathrm{IKE}\right)\times 100\% \end{array}$$

The HPLC method was as described in the previous study.^[^ ^67^ ^]^

### Characterization and Stability of TDN and TDN‐IKE

The synthesis of TDN was assessed using 3% agarose gel electrophoresis. The Zeta potentials and particle size were measured using DLS (dynamic light scattering) with a Nano ZS instrument (Malvern, UK). The shapes and sizes of tFNA‐Cur were determined using AFM (atomic force microscopy). In stability experiments, TDN and TDN‐IKE were separately incubated in 5% (v/v) fetal bovine serum (FBS) at 37 °C for various times (0, 3, 6, 12, 24, and 48 h). The stability of TDN/TDN‐IKE was assessed using 3% agarose gel electrophoresis. AFM (MFP‐3D Origin, Oxford Instruments) and TEM (Talos F200i TEM, Thermo Fisher) were used to measure the sizes and shapes of TDN and TDN‐IKE. For AFM, samples were prepared on the silicon wafer. AFM imaging was performed using the tapping mode under a 0.6N/M cantilever spring constant in air. For TEM, samples (10 µL) were spotted on an ultrathin microgrid (copper mesh), and the samples were dried naturally. Then, the morphology analysis of the samples was characterized by TEM. Three fields of view were used, and three independent repeated experiments were performed.

### Cell Viability Effects of TDN

LN229 cells were seeded into 96‐well plates with 3000 cells per well. The cells were treated with different concentrations (0, 25, 50, 100, 200 nM) of TDN for 24 h. After a 90 min incubation with CCK‐8 solution (MCE, USA), the cell viability was assessed by measuring the optical density (OD) value at 450 nm.

### Cellular Uptake of TDN and TDN‐IKE

LN229 cells were cultured in confocal dishes and then co‐incubated with FAM‐labeled TDN/TDN‐IKE (200 nM). After 4 h, cells were stained with Hoechst (Beyotime), and a confocal laser microscope (Zeiss, Germany) was used to observe the TDN/TDN‐IKE uptake images of cells. For flow cytometry, the cells were seeded in a 12‐well plate and treated with free FAM, FAM‐labeled TDN, or TDN‐IKE (200 nM). After 4 h, cells were harvested, and the relative fluorescence intensity of Cy5 was detected using a flow cytometer (Qinxiang, China). An unstained control was used for gating and baseline mean fluorescence intensity.

### Brain Distribution of TDN in Mice

To determine the BBB capacity of TDN, Cy5‐labeled TDN were administered to mice via tail intravenous injection. Mice were anesthetized and subjected to time‐tracking imaging with a small‐animal in vivo imaging system (Tanon, China) at various time points after injection (5, 10, 15, 20, 25, 30, and 60 min).

### TDN‐Mediated IKE Brain Tissue Release

An orthotopic xenograft tumor model was established as described above, and 100 µL TDN‐IKE (20 mg kg^−1^) was intravenously injected into model mice. Two hours later, the mice were euthanized, and brain tumor tissue and healthy tissue were collected and quickly frozen in liquid nitrogen for IKE detection. Each 40 mg brain tissue sample was added to 100 µL of PBS, ground thoroughly using an organizational grinder, and then transferred to a 1.5 mL tube. Next, 900 µL of acetonitrile was added, followed by thorough mixing and centrifugation to collect the supernatant. After freeze‐drying, the samples were dissolved in methanol for liquid chromatography mass spectrometry (Thermo, TSQ Altis) analysis, and IKE concentrations were calculated.

### In Vitro Hemolysis Assay

Blood was collected from nude mice in EDTA‐coated tubes and centrifuged at 4000 rpm for 5 min to remove the upper plasma. Then, the red blood cell pellet was washed three times with cold PBS and diluted 1/10 with cold PBS. TDN was added to the red blood cell suspension at concentrations of 0, 100, 200, 400, 600, 800, and 1000 nm. Phosphate buffer (PBS) and 0.1% TritonX‐100 were used as negative and positive controls, respectively. After incubation at 37 °C for 4 h, the solution was centrifuged at 4000 rpm for 10 min, and the OD of 200 µL of the supernatant was measured at 545 nm. The percentage of hemolysis was calculated using the following formula:

(2) $$\begin{array}{ccc} \mathrm{hemolysis}\left(\%\right) & = & \left(OD_{\mathrm{test}}-OD_{\mathrm{negtive}\;\;\mathrm{control}}\right)/\\ & & \times \left(OD_{\mathrm{positive}\;\;\mathrm{control}}-OD_{\mathrm{negative}\;\;\mathrm{control}}\right)\times 100\% \end{array}$$

### In Vivo Toxicity Assessment

Fourteen female nude mice (6–8 weeks) were divided into two groups (7 mice per group) and given PBS or TDN‐IKE intravenously every other day for overall 12 times. The mice were sacrificed on day 24, and their blood samples were collected and delivered to Wuhan Servicebio Technology Co., Ltd for blood biochemical evaluations.

### Data Acquisition and Preprocessing

RNA sequencing data and corresponding clinical information were obtained from the Chinese Glioma Genome Atlas (CGGA) database (http://www.cgga.org.cn). Patients lacking overall survival (OS) time or PDIA3 expression data were excluded from the analysis. After filtering, a total of 608 patients with complete clinical and molecular information were included. PDIA3 expression was divided into high and low groups according to the median value. The clinical variables included WHO grade, IDH mutation status, patient age, and recurrence status. All statistical analyses were conducted using R software (version 4.5.1; R Foundation for Statistical Computing, Vienna, Austria). The main R packages used included “survival,” “survminer,” “ggplot2,” “dplyr,” and “tidyr.”

### RNA Sequencing

Total RNA was extracted using the TRIzol reagent (Invitrogen, CA, USA) according to the manufacturer's protocol. RNA purity and quantification were evaluated using the NanoDrop 2000 spectrophotometer (Thermo Scientific, USA). RNA integrity was assessed using the Agilent 2100 Bioanalyzer (Agilent Technologies, Santa Clara, CA, USA). Then the libraries were constructed using VAHTS Universal V6 RNA‐seq Library Prep Kit according to the manufacturer's instructions. The transcriptome sequencing and analysis were conducted by OE Biotech Co., Ltd. (Shanghai, China).

### Statistical Analysis

Statistical analysis was performed using GraphPad Prism 8.0 software (LaJolla, USA). All values are reported as the mean ± standard deviation. All tests performed were two‐sided. Comparisons between two groups were performed by unpaired Student's *t*‐test, and one‐way analysis of variance and two‐way analysis of variance with Student–Newman–Keuls test were employed for comparisons among multiple groups. *p* < 0.05 was considered to indicate statistical significance.

## Conflict of Interest

The authors declare no conflict of interest.

## Author Contributions

J.Z., W.W., X.L., P.L., and X. L. contributed equally to this work, performed the experiments. Y.W., Q.M., T.X., and J.J. conceived the project. X.L., W.W., J.Z., P.L., and X.L. performed experiments and helped analyze data with assistance from Q.N., X.Z., H.L., D.Y., and L.S. W.L., and K.L. provided suggestions for the project design. Animal experiments were performed by X.L., W.W., X.L., and P.L. S.X., X.H., S.Z., and W.C. performed gene expression and RNA‐seq analysis. Y.W., Q.M., J.Z., and J.J. wrote an original manuscript. The manuscript was edited, and the intellectual inputs were given by all authors. Y.W. and T.X. supervised the project. The author(s) read and approved the final manuscript.

## Supporting information



Supporting Information

Supporting Information

Supporting Information

Supporting Information

Supporting Information

## Data Availability

The data that support the findings of this study are available in the supplementary material of this article.
