# Supplementary material for: PDIA3 Inhibition Facilitates Sensitivity of IKE‐Induced Ferroptosis via STAT3/LCN2 Axis to Improve Glioblastoma Therapy
Source: Adv Sci (Weinh). 2025 Dec 14;13(12):e14191. doi: 10.1002/advs.202514191 (PMC12948197; doi:10.1002/advs.202514191)
Supplement: Supplementary file 1 — Supporting Information [file ADVS-13-e14191-s003.docx]

**Figure S1 PDIA3 is a potentially key PDI family gene that regulates IKE and cystine starvation-induced ferroptosis**


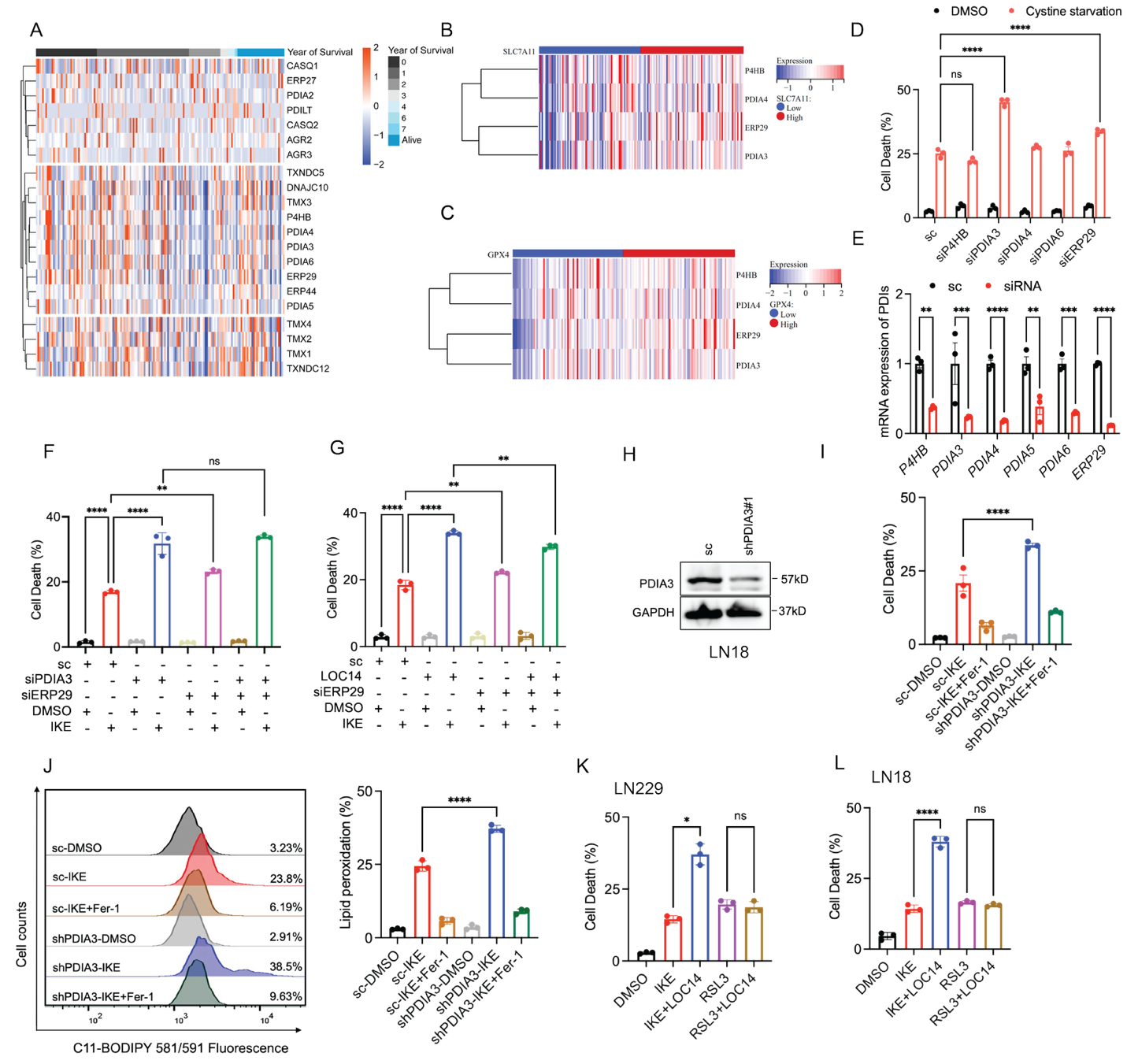


(A) Associations between the expression of PDI family genes and survival of GBM patients in TCGA GBM dataset. (B) Associations between the indicated candidate genes and GPX4 in TCGA GBM dataset. (C) Associations between the indicated candidate genes and SLC7A11 in TCGA GBM dataset. (D) Detection of cell death using PI through flow cytometry in LN229 cells transfected with indicated siRNAs and scrambled control (sc) as under cystine starvation conditions for 24h. (E) KD efficiency of PDI family genes as tested by qRT-PCR. (F) Quantitative analysis of cell death via flow cytometry in LN229 cell transfected with the indicated siRNAs and sc as control after IKE treatment. (G) Quantitative analysis of cell death via flow cytometry in LN229 cell transfected with siRNA targeting ERP29 and sc as control after IKE treatment combined with LOC14. (H) PDIA3 protein levels in LN18 PDIA3-sh cells as detected by western blot analysis. (I) Quantitative analysis of cell death via flow cytometry following IKE treatment in LN18 shPDIA3 cells. (J) Representative flow cytometry and quantitative analysis of lipid peroxidation levels by flow cytometry after IKE treatment of PDIA3-sh cells. (K) Quantification of LN229 cells via flow cytometry after IKE and RSL3 combined with LOC14, respectively. (L) Quantitative analysis of LN18 cells treated with IKE and RSL3 combined with LOC14 by flow cytometry. The results are presented as mean ± SD, with significance levels indicated as follows, **P*<0.05, ***P*<0.01, ****P*<0.001, *****P*<0.0001.

**Figure S2 PDIA3 inhibition increases ferroptosis sensitivity caused by cystine starvation in GBM**


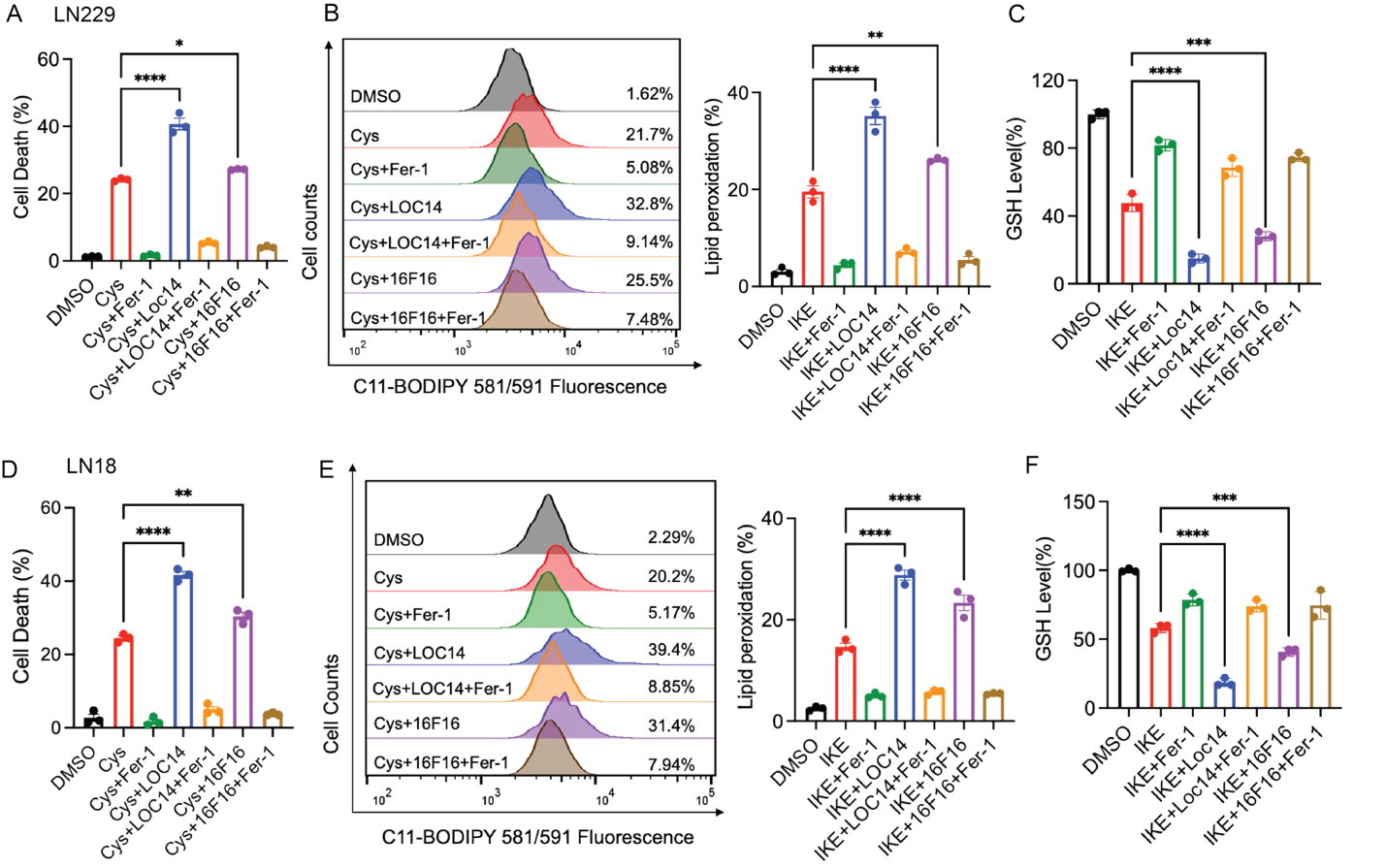


(A) Quantitative analysis of cell death detected by flow cytometry after treatment with PDIA3 inhibitors 16F16 and LOC14 in combination with cystine starvation in LN229 cells. (B) Representative flow cytometry and quantitative analysis of lipid peroxidation levels by flow cytometry after treatment of LN229 cells with 16F16 and LOC14 combined with cystine starvation. (C) GSH levels were detected after treatment with PDIA3 inhibitors 16F16 and LOC14 in combination with IKE in LN229 cells. (D) Quantitative analysis of cell death by flow cytometry after treatment with PDIA3 inhibitors 16F16 and LOC14 in combination with cystine starvation in LN18 cells. (E) Representative flow cytometry plots and quantification of lipid peroxidation levels by flow cytometry after cells treated with 16F16 and LOC14 combined with cystine starvation in LN18 cells. (F) GSH levels in LN229 cells were detected after treatment with PDIA3 inhibitors 16F16 and LOC14 in combination with IKE. The mean SD was used to express the results, **P*<0.05, **P*<0.01, ****P*<0.001, *****P*<0.0001.

**Figure S3 PDIA3 stability is regulated by E3 ubiquitin ligase NEDD4L and necessary for IKE-induced ferroptosis**


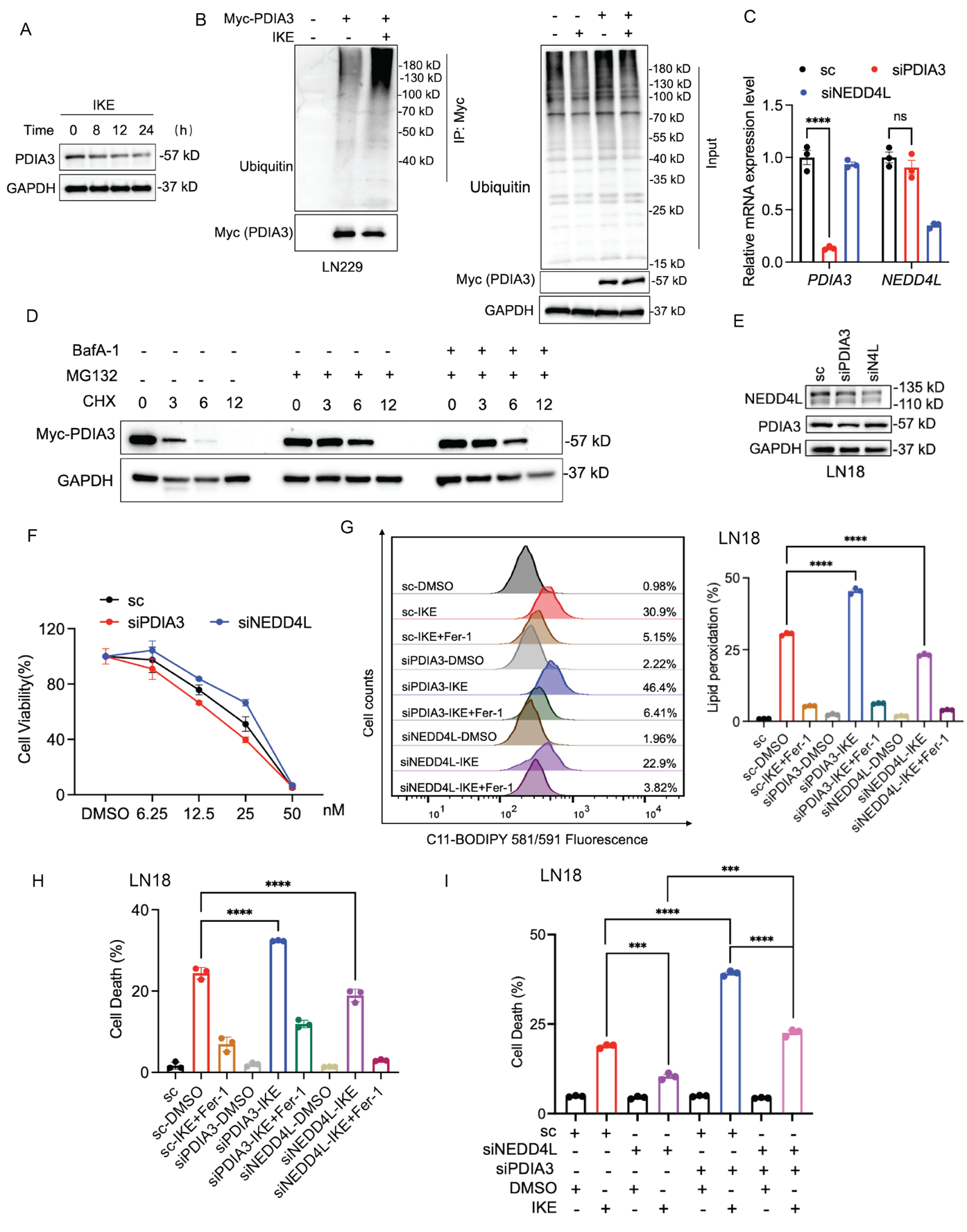


(A) Protein levels of PDIA3 in LN229 cells treated for various times. (B) Ubiquitination of PDIA3 was detected by co-immunoprecipitation and western blotting, LN229 cells were transfected with the indicated plasmids under IKE treatment for 12 hours. (C) mRNA levels of *PDIA3* and *NEDD4L* as detected by qRT-PCR in LN229 cells transfected with the indicated siRNAs. (D) Western blot detection of the indicated proteins in 293T cells transfected with the indicated plasmids and treated with or without MG132±BafA1 by CHX assay, according to the time gradient. (E) Protein levels of PDIA3 and NEDD4L was detected by western blotting in LN229 cells transfected with the indicated siRNAs. (F) Cell viability after transfection of LN229 cells with the indicated siRNAs under treatment with different concentrations of IKE. (G) Detection and quantitative analysis of lipid peroxidation level via flow cytometry in LN18 cells transfected with the indicated siRNAs. (H) Detection and quantitative analysis of cell death via flow cytometry in LN18 cells transfected with the indicated siRNAs. (I) Detection and quantitative analysis of cell death via flow cytometry in LN18 cells transfected with the indicated siRNAs. The result is expressed as mean ± SD, ****P*<0.001, *****P*<0.0001, ns: no significance.

**Figure S4 NEDD4L facilitates K29-branched ubiquitination degradation of PDIA3**


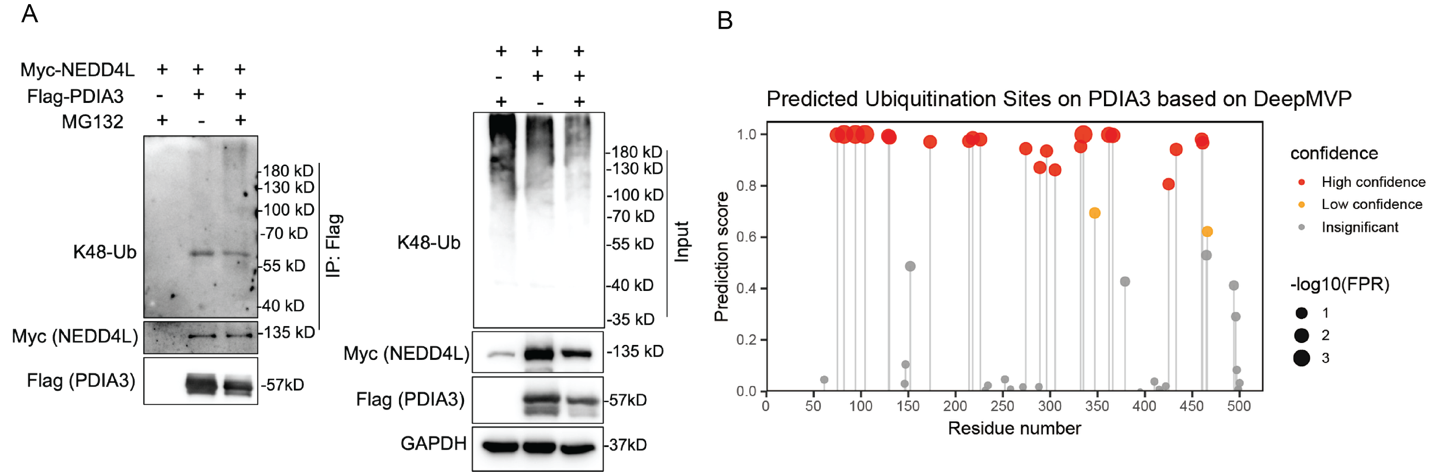


(A) K48 branched ubiquitination of PDIA3 was detected by co-immunoprecipitation and western blotting, 293T cells were transfected with the indicated plasmids. (B) Mapping of multiple potential ubiquitination sites which are predicted by DeepMVP software.

**Figure S5** **PDIA3 mediates ferroptosis sensitivity via STAT3/LCN2 axis**


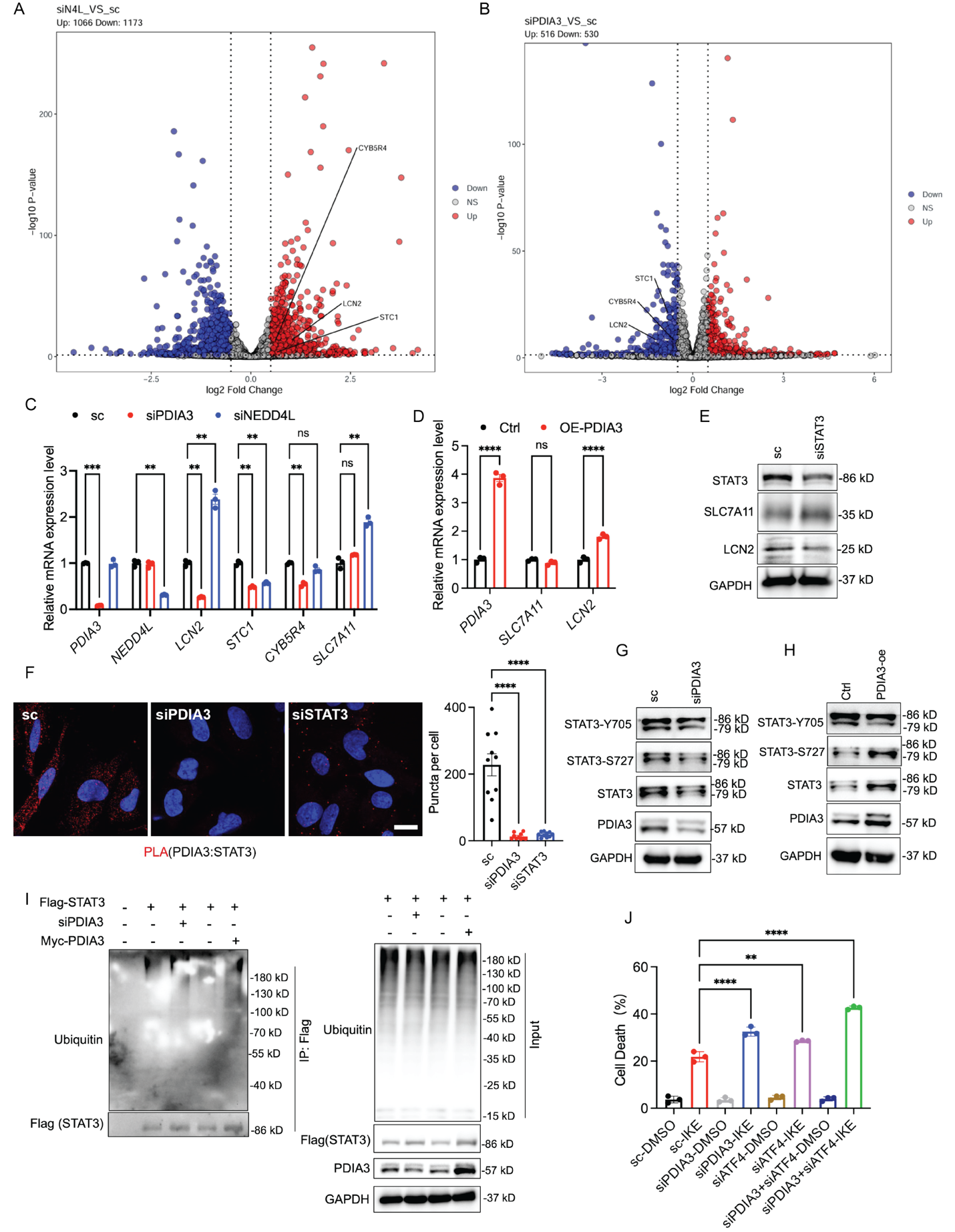


(A, B) Volcano plots representing differentially expressed genes (upregulated and downregulated genes are indicated) in LN229 siNEDD4L (A) or siPDIA3 cells (B) compared to control cells.

(C) qRT-PCR analysis of mRNA expression levels of *LCN2*, *STC1*, *CYB5B4*, *SLC7A11*, *PDIA3*, and *NEDD4L* in LN229 cells transfected with the indicated siRNAs. (D) Expression levels of *SLC7A11* and *LCN2* mRNA were detected in LN229 cells transfected with the indicated plasmids. (E) Expression levels of SLC7A11 and LCN2 protein were detected by western blotting in LN229 cells transfected with the indicated plasmids and siRNAs. (F) Interaction between PDIA3 and STAT3 was observed by proximity ligation (PLA) after siRNA transfection into LN229 cells, PLA signals (dots) in each cell were quantified. PLA signals (dots) in each cell were quantified. Two-way ANOVA. *****P* < 0.0001. Each data point corresponds to an image field that contains an average of 10 cells. n = 6–8 images in each condition as indicated. All images were collected from one experiment. Two independent experiments were performed, with similar results. Scale bar: 20 μm. (G, H) Expression levels of STAT3 and p-STAT3 protein were detected by western blotting in LN229 cells transfected with the indicated siRNAs and plasmids. (I) Ubiquitination of STAT3 was detected by Flag co-immunoprecipitation and western blotting, 293T cells were transfected with the indicated plasmids. (J) Detection and quantitative analysis of cell death via flow cytometry under 25 μM IKE treatment for 24 hours in LN229 cells subjected to the indicated siRNAs. The results are presented as mean ± standard deviation (SD), with n = 3, ***P*<0.01, *****P*<0.0001 and ns indicating no significance.

**Figure S6 Survival analysis with respect to PDIA3 expression status in CGGA samples**

**
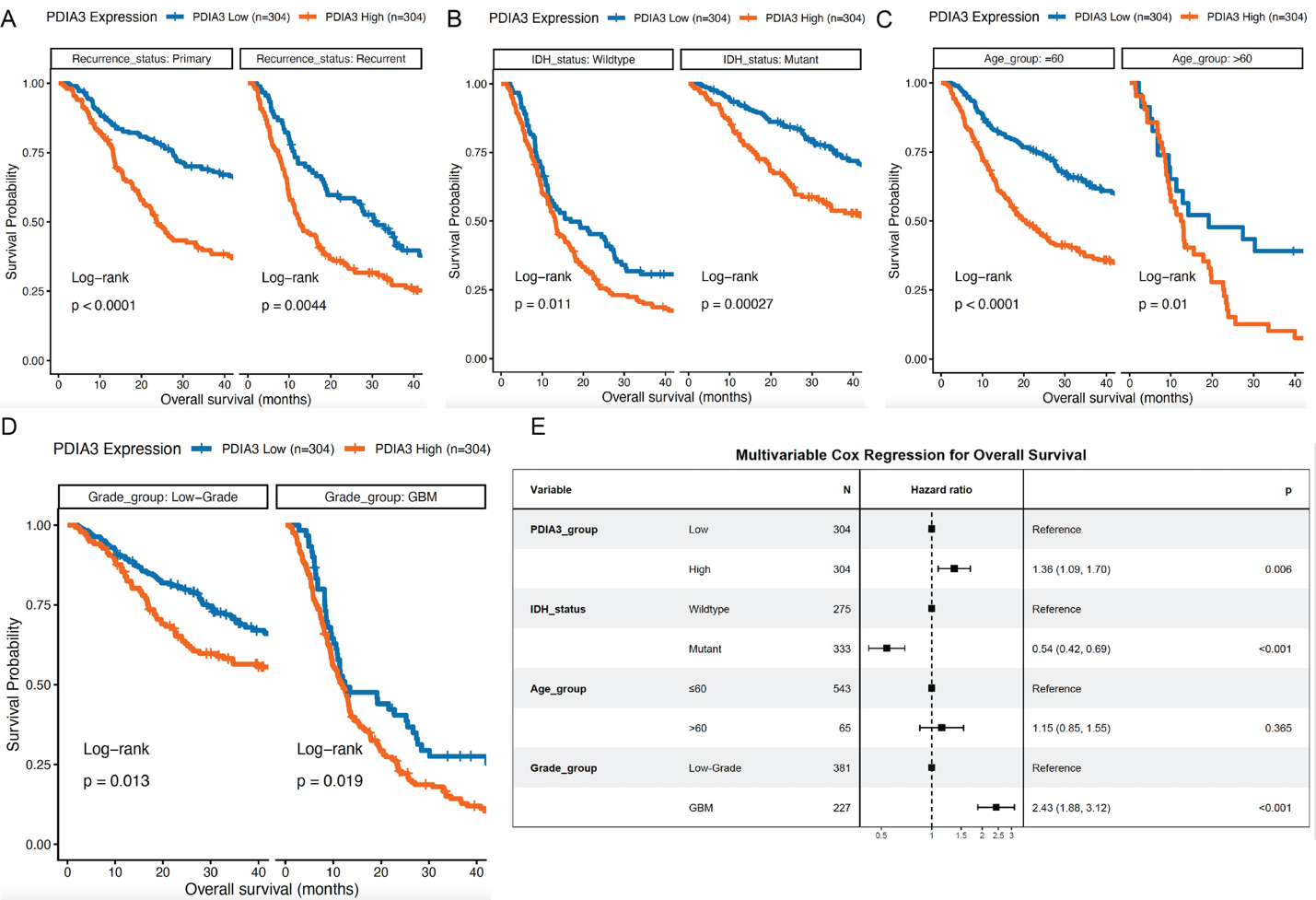
**

(A-D). Kaplan–Meier (KM) survival curves of the high- and low-expression groups according to recurrence (A), IDH mutation (B), patient age (C), and WHO grade (D) status in CGGA datasets. (E) Multivariable cox proportional hazards regression analysis for overall survival including PDIA3 expression group, patient age, IDH mutation status, and WHO grade group.

**Figure S7 Characterization of TDN and TDN-IKE**


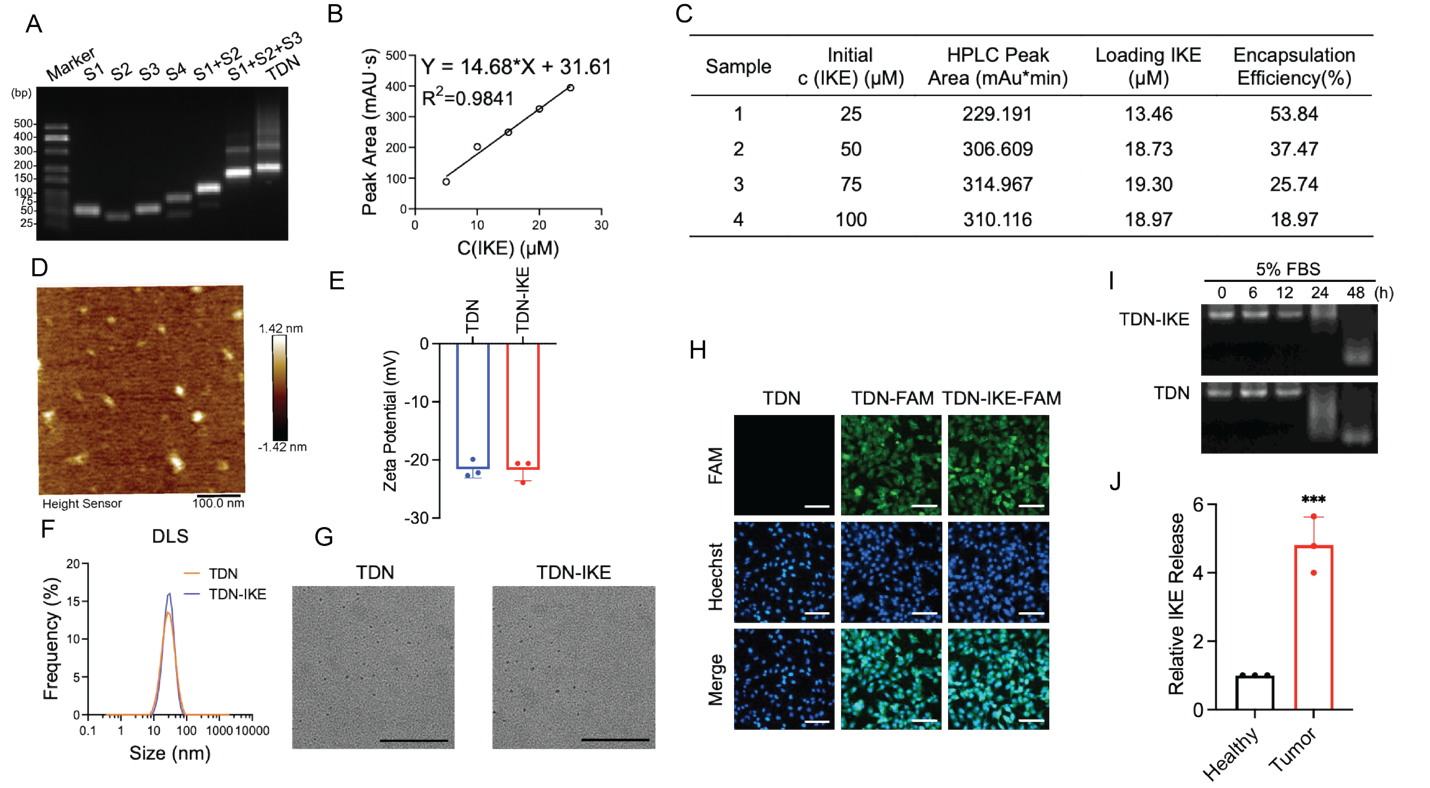


(A) Agarose gel showing successful synthesis of TDN. (B, C) Standard curve of IKE (B) and encapsulation efficiency of IKE into TDN (C). (D) AFM image of TDN-IKE. (E) Zeta potential of TDN / TDN-IKE. (F) Partial size of TDN/TDN-IKE. (G) TEM images of TDN and TDN-IKE, scale bar: 50 nm. (H) confocal microscopy imaging of uptake of TDN/TDN-IKE in LN229 cells. Scale bars, 20 μm. (I) Serum stability of TDN / TDN-IKE in 5% FBS at 37 °C for 0-48 h. (J) Relative IKE release of brain GBM tissue and healthy tissue 2 h after intravenous injection of TDN-IKE into GBM orthotopic xenograft tumor model. The results are presented as mean ± standard deviation(SD)，with n = 3, ****P*<0.001.

**Figure S8 Biosafety evaluation *in vitro* and *in vivo***


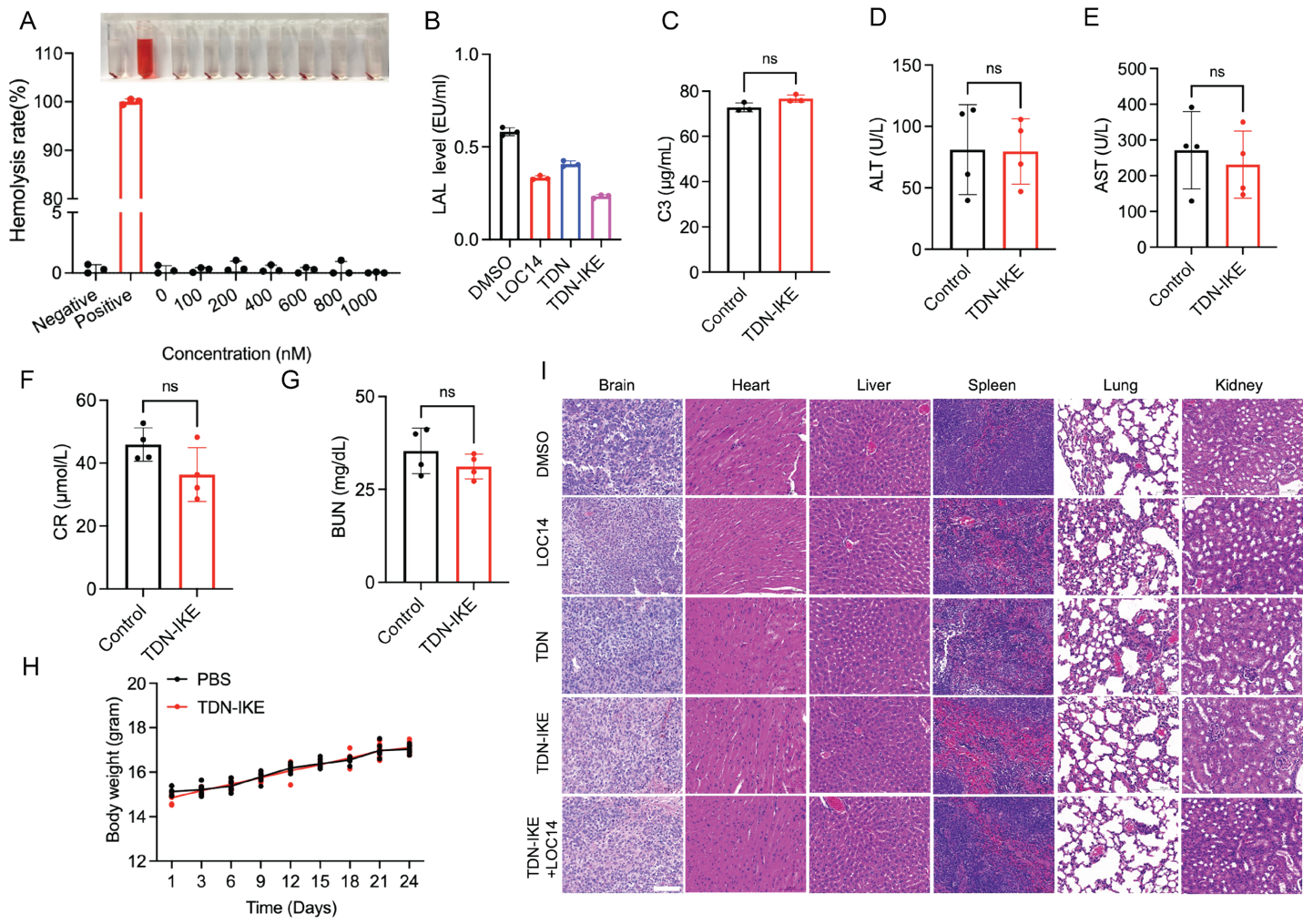


(A) *In vitro* hemolysis of TDN at different concentrations. Negative control: PBS, Positive control: 0.1% TritonX-100. (B) Test of endotoxin levels for the indicated groups using Limulus Amebocyte Lysate (LAL) assay. (C) Measurement of C3 protein for complement activation in the indicated groups. (D-G) Measurement of blood serum biochemistry for liver and kidney function parameters. ALT alanine aminotransferase, AST aspartate aminotransferase, CR creatinine, BUN blood urea nitrogen. Data are presented as mean values ± SD. ns indicating no significance. (H). Body weights of mice in the indicated groups. (I) Hematoxylin and eosin (H&E) staining of the major organs after drug treatment. Scale bar: 100 μm.
